# Mitotic Hub Gene Network in Colorectal Cancer: Integrated Transcriptomic, Protein-Level, and Clinical-Genomic Characterization of a Ten-Gene Signature

**DOI:** 10.3390/genes17070783

**Published:** 2026-07-08

**Authors:** Ebtihal Kamal, Ehssan Moglad, Samah O. Mohager, Mehad Ahmed, Mobarak Mahfod Aldoseri, Barakat A. Al Suwayyid, Azizah Salim Bawadood, Hamdan Z. Hamdan, Mikail Akbulut

**Affiliations:** 1 Department of Basic Medical Sciences, College of Medicine, Prince Sattam bin Abdulaziz University, Al-Kharj 16273, Saudi Arabia; s.hamadain@psau.edu.sa (S.O.M.); ma.ahmed@psau.edu.sa (M.A.); b.alsuwayyid@psau.edu.sa (B.A.A.S.); a.bawadood@psau.edu.sa (A.S.B.); 2 Department of Pharmaceutics, College of Pharmacy, Prince Sattam bin Abdulaziz University, Al-Kharj 16273, Saudi Arabia; e.moglad@psau.edu.sa; 3 College of Medicine, Prince Sattam bin Abdulaziz University, Al-Kharj 16273, Saudi Arabia; mobarakondemand@gmail.com; 4 Department of Pathology, College of Medicine, Qassim University, P.O. Box 6655, Buraidah 51452, Saudi Arabia; h.abualbasher@qu.edu.sa; 5 Department of Biology, Faculty of Science, Erciyes University, Kayseri 38280, Türkiye; akbulut@erciyes.edu.tr

**Keywords:** colorectal cancer, prognostic markers, bioinformatics, differentially expressed genes, machine learning

## Abstract

**Background**: Colorectal cancer (CRC) remains a heterogeneous disease, and improved biomarkers are needed to support prognostic assessment. This study aimed to characterize hub genes in CRC and evaluate whether a gene signature provides biologically meaningful and prognostic information in clinical–genomic models. **Methods**: We integrated three GEO microarray datasets (GSE110223, GSE110224, and GSE23878) to identify common differentially expressed genes using adjusted p<0.05 and $|{log}_{2}FC|>1$. Hub genes and protein expression were identified through protein–protein interaction network analysis using maximal clique centrality and Human Protein Atlas, respectively. Prognostic relevance was evaluated in TCGA-COAD/READ using Kaplan–Meier analysis, multivariable Cox regression, Cox-derived prognostic indices, time-dependent ROC analysis, and regression-based machine learning for internal robustness. Principal component analysis (PCA) was used to derive a standardized PC1-based score from the 10-hub gene signature. **Results**: A ten-gene mitotic hub signature (TPX2, UBE2C, AURKA, NEK2, PRC1, CCNB1, CDK1, CEP55, FOXM1, and RRM2) was consistently upregulated across the three datasets and enriched for cell-cycle and mitotic pathways. Protein-level and survival analyses supported the biological relevance of several hub genes. In TCGA-COAD/READ, the signature showed limited standalone prognostic value and did not retain independent significance after adjustment for clinical variables, although it contributed modestly in integrated clinical–genomic models. PCA showed a one-dimensional signature, with PC1 capturing the dominant shared expression pattern. Gradient Boosting Regressor (R^2^ = 0.8035, MSE = 0.0473) supported the internal robustness of the DEG-based expression pattern. **Conclusions**: The ten-gene mitotic hub signature represents a coherent CRC-related proliferative program with limited value as an isolated prognostic marker, but it may still be useful as part of integrated risk models that require external validation.

## 1. Background

Colorectal cancer (CRC) is one of the most common cancers worldwide, with millions of new cases every year, making it a leading cause of cancer-related mortality globally [1,2]. Despite the availability of effective screening tools such as colonoscopy, early detection remains limited by the lack of reliable molecular biomarkers [3]. CRC is characterized by considerable biological, histological, and molecular heterogeneity caused by various genetic and epigenetic changes, which complicate prognostic evaluation and contribute to variable clinical outcomes [4,5]. Despite the remarkable progress in the current therapeutic strategies, survival rates remain low due to late diagnosis and limitations in current diagnostic approaches [6].

The tumor, node, and metastasis (TNM) classification remains the standard framework for CRC prognostication, but it does not capture the molecular diversity that drives tumor progression, recurrence, and survival outcomes [7,8]. Consequently, there is a need for robust molecular markers that can improve biological understanding and support more refined risk stratification. Gene expression profiling offers a useful approach to identify the dysregulated genes involved in CRC pathogenesis and differentially expressed genes (DEGs), providing insight into candidate biomarkers and disease mechanisms. These molecular signatures are promising for targeted therapeutic approaches in precision medicine for CRC [8]. Mitotic dysregulation constitutes one hallmark of cancer, leading to chromosomal instability, proliferative independence, and tumor aggressiveness [9,10]. In solid tumors such as CRC, genes involved in spindle assembly, checkpoint control, and mitotic progression are commonly altered in CRC, indicating that mitosis-focused signatures may reflect biologically relevant features of tumor behavior [11]. Therefore, a mitosis-oriented signature may shed biological insight into tumor behavior and may have prognostic value if tested in the right clinical setting [12]. Large-scale expression datasets have been increasingly analyzed using bioinformatics and machine learning approaches to identify molecular signatures through differential expression analysis, functional enrichment analysis, protein–protein interaction network mapping, and model-based evaluation of candidate genes [13]. While these approaches have generated numerous candidate signatures, many lack sufficient validation or are interpreted beyond their evidentiary scope. Recent advances in the field of colorectal cancer have been increasingly based on transcriptomic profiling and integrative bioinformatics to find prognostic biomarkers and to elucidate disease-related molecular mechanisms. Gu et al. developed a prognostic model based on mitosis-related gene signatures from The Cancer Genome Atlas (TCGA) and Gene Expression Omnibus (GEO) cohorts [14]. Li et al. and Guo et al. identified common hub genes, including AURKA, CCNB1, and TPX2, through differential expression analysis and network-based screening [15,16]. Most recently, Wang further constructed a multi-omics risk model using integrative approaches and performed machine learning methods [17]. While these studies highlight the potential of transcriptomic signatures, they primarily emphasize prognostic model construction rather than the biological coherence of underlying gene networks. The purpose of this study was to characterize a mitotic hub-gene program in colorectal cancer and assess its coherence across independent transcriptomic datasets. Unlike prior CRC studies that primarily emphasized prognostic score construction, the present work focuses on the biological characterization of a CRC-associated mitotic hub-gene program. By integrating independent transcriptomic datasets, protein-level evidence, protein–protein interaction network analysis, pathway enrichment, protein-level assessment, survival, and internal robustness evidence through machine learning. This distinction is practically important because it helps define whether such signatures should be interpreted as mechanistic biological markers, prognostic tools, or both. This framing distinguishes biomarker characterization from prognostic tool development and clarifies the scope of the present work.

To our knowledge, this study differs from previous CRC gene-signature reports by focusing specifically on mitotic hub genes and by distinguishing their biological relevance from their prognostic performance.

## 2. Methods

### 2.1. Data Acquisition and Preprocessing

Microarray gene expression profiles associated with colorectal cancer (CRC) were obtained from the Gene Expression Omnibus (GEO), a public functional genomics repository hosted by the National Center for Biotechnology Information (NCBI) [18]. We included human studies comparing colorectal adenocarcinoma tissues with matched adjacent non-cancerous tissues. Three GEO microarray datasets, GSE110223 (PMID: 30809322), GSE110224 (PMID: 30809322), and GSE23878 (PMID: 21281787), were selected for the discovery analysis based on predefined inclusion criteria. We prioritized these datasets as they contained an adequate number of colorectal tumors and corresponding normal control samples, provided access to raw expression files, and were generated on Affymetrix platforms suitable for consistent preprocessing and gene-level harmonization. The use of these datasets enabled uniform normalization within each cohort and facilitated cross-dataset comparison for robust differential-expression analysis. This selection strategy was intended to maximize the reliability and reproducibility of the discovery-phase results while minimizing bias introduced by incomplete data availability or incompatible preprocessing requirements. A description of the datasets used in this study was presented in Table S1.

Raw CEL files for GSE110223 (Affymetrix GPL96 platform) and GSE110224 and GSE23878 (Affymetrix GPL570 platform) were downloaded and processed in R (v4.3.0) using Bioconductor packages [19]. Robust Multi-array Average (RMA) normalization was applied separately to each dataset to minimize technical variation within datasets and to account for platform-specific probe behavior. Probes were mapped to official gene symbols, and only genes represented consistently across platforms were retained for downstream analysis. Gene-level expression matrices were then harmonized for comparative analysis. Because the datasets were generated on different Affymetrix platforms, no additional empirical batch correction was applied to avoid over-adjustment and potential loss of true biological signal.

### 2.2. Differential Expression Analysis

Differential expression analysis between paired tumor and normal samples was performed using the limma package in R (version 4.5.1) [20]. Linear modeling and empirical Bayes moderation were used to identify differentially expressed genes (DEGs). The criteria for DEG selection as significant genes were an adjusted *p*-value of less than 0.05 and |log_2_ fold change| > 1. DEGs across the three datasets were visualized using Venny 2.1 (https://bioinfogp.cnb.csic.es/tools/venny/ accessed on 4 April 2026). The significantly expressed genes were classified as upregulated for log_2_-fold change > 1 and downregulated for log_2_-fold change < −1. Volcano plots were generated using ggplot2 to visualize fold change (represented on the *x*-axis) versus statistical significance (represented by the negative log_10_-transformed adjusted *p*-value on the *y*-axis) [21]. Heatmaps of DEGs were generated using the pheatmap package in R [22]. Expression values were standardized by z-score transformation to facilitate comparison across genes, and hierarchical clustering was used to group both genes and samples.

### 2.3. Functional Enrichment Analysis

Functional enrichment analysis of co-DEGs was performed using Enrichr (https://maayanlab.cloud/Enrichr/ accessed on 20 April 2026), an efficient web-based comprehensive functional gene analysis platform for gene set enrichment analysis [23]. Gene Ontology (GO) analysis was conducted across three categories: Biological Process, Molecular Function, and Cellular Component. Kyoto Encyclopedia of Genes and Genomes (KEGG) pathway enrichment analysis was also performed to identify significantly overrepresented pathways. Enrichment terms with a *p*-value ≤ 0.05 were considered significant.

### 2.4. PPI Network Construction and Hub Gene Identification

The co-DEGs were submitted to the STRING database (version 12.0) to construct a protein–protein interaction (PPI) network [24]. Interactions were queried for Homo sapiens using co-expression, experimental evidence, curated databases, and text mining, with a minimum interaction score of 0.7. The resulting network was imported into Cytoscape (version 3.10.1) for visualization and topological analysis. Hub genes were identified using the CytoHubba plugin in Cytoscape. Maximal Clique Centrality (MCC) was used to rank nodes by network connectivity, and the top 10 genes were selected as hub genes [25].

### 2.5. Assessment of Protein Expression

The Human Protein Atlas (HPA) (https://www.proteinatlas.org/ accessed on 5 May 2026) charts all human proteins across cells, tissues, and organs by integrating a range of omics-based technologies. It also utilizes various histological methods, such as antibody-based imaging, proteomics via mass spectrometry, and transcriptomics [26]. Protein expression patterns of the selected hub genes were evaluated using the HPA. Immunohistochemistry images for normal and colorectal tumor tissues were downloaded from the HPA and visually compared to assess consistency with transcriptomic findings. The antibodies used included HPA005487, CAB001454, CAB017530, HPA034521, CAB000115, HPA023430, CAB017832, HPA056994, and CAB080407.

### 2.6. Evaluation of Mitotic Hub Score Distribution in TCGA-COAD/READ Tumor and Normal Samples

TCGA-COAD/READ gene-expression data were used to calculate a mitotic hub score based on the mean expression of ten hub genes, including TPX2, UBE2C, AURKA, NEK2, PRC1, CCNB1, CDK1, CEP55, FOXM1, and RRM2. Samples were classified as normal or tumor using the TCGA barcode field, and the hub score was compared between the two groups. Group differences were assessed visually using a boxplot and statistically using both the Wilcoxon rank-sum test and one-way ANOVA.

### 2.7. Correlation Analysis Between Mitotic Hub Score and Proliferation Score

A proliferation score was calculated as the mean expression of MKI67, TOP2A, PCNA, and BIRC5. The two scores were then correlated across samples, and summary statistics were generated by cancer type.

### 2.8. Survival Analysis and Prognostic Evaluation

#### 2.8.1. Overall Survival Analysis by Mitotic Hub Gene Risk Group

Overall survival (OS) analysis was performed in the full TCGA-COAD/READ cohort and in the stage II–III subset. OS time (days) and vital status (alive or dead) were obtained from TCGA clinical annotations, with OS defined as the interval from diagnosis to death from any cause or last follow-up. A continuous mitotic hub-gene risk score was calculated for each patient as the standardized mean expression of the ten hub genes after mapping Ensembl identifiers to HGNC symbols. The risk score was merged with clinical annotations using TCGA patient barcodes. Patients were dichotomized into high- and low-risk groups using the median risk score as the cutoff. Kaplan–Meier survival curves were generated using the survfit function in the survival package, and between-group differences were assessed using the log-rank test. Survival curves and numbers at risk were plotted using ggsurvplot in the survminer package.

#### 2.8.2. Multivariable Cox Analysis of the Mitotic Hub Gene Signature

To evaluate the independent prognostic value of the mitotic hub-gene signature, multivariable Cox proportional hazards models were fitted with OS as the outcome and the continuous risk score as the primary predictor, adjusting for age at diagnosis, sex, and pathological stage. Hazard ratios with 95% confidence intervals and Wald *p*-values were reported for each covariate. Model performance was assessed using likelihood ratio, Wald, and score tests, and discrimination was quantified using the concordance index. For Cox-derived prognostic stratification, multivariable Cox models were also fitted in the full TCGA-COAD/READ cohort and in the stage II–III subset. Individual prognostic indices were calculated as weighted linear predictors from the fitted models. Patients were then assigned to low-, intermediate-, or high-risk groups according to tertiles of the prognostic index. Overall survival by risk group was evaluated using Kaplan–Meier analysis, with log-rank testing and estimation of 1-, 3-, and 5-year OS probabilities. Median OS and corresponding 95% confidence intervals were extracted from the survival curves.

#### 2.8.3. Time-Dependent ROC Analysis of the Mitotic Hub Gene Signature in the TCGA Cohort

Time-dependent receiver operating characteristic (ROC) analysis was performed in the TCGA-COAD/READ cohort and the stage II–III subset using the timeROC package in R. Cumulative/dynamic ROC curves were generated for 1-, 3-, and 5-year OS using the continuous risk score, and the corresponding areas under the curve (AUCs) were calculated to quantify prognostic discrimination over time.

#### 2.8.4. Principal Component Analysis-Derived Signature Score

A principal component analysis (PCA) was performed on the expression matrix of the 10-hub genes using z-scored gene expression values per sample to ensure each gene contributed on a comparable scale. The principal component, which captured the largest proportion of variance in the gene set (PC1), was extracted for each sample and standardized to have a mean of 0 and unit variance, yielding the PCA-based signature score, signature_score_pca_z.

##### Association with Survival Status and Overall Survival by PCA Score

To assess whether the PCA-based signature score differed by outcome, we linked the PC1-derived scores to clinical data via exact TCGA barcode matching and grouped patients by vital status (Alive vs. Dead). A box plot was generated to display the distribution of signature_score_pca_z in the two survival-status groups. Overall survival was analyzed using the Kaplan–Meier method, and patients were stratified into low and high PCA signature score groups. Survival curves were compared using the log-rank test, and the number at risk was displayed at selected time points to summarize follow-up. The PCA score grouping was used to evaluate whether the molecular signature was associated with differences in survival probability over time.

##### Association with the Pathological Stage by PCA Score

To evaluate the relationship between the PCA-based signature and disease stage, the same standardized PC1 scores were merged with the pathologic stage data. The stage was categorized into sub-stages (Stage I, IA, II, IIA, IIB, IIC, III, IIIA, IIIB, IIIC, IV, IVA, and IVB). The distribution of signatures across the stage categories was visualized using a violin plot with boxplots, showing the central tendency and spread of scores in each stage. To estimate hazard ratios for the association between clinicopathologic stage and survival risk, a Cox proportional hazards model, with stage I as the reference category, was performed. Results are reported as hazard ratios with 95% confidence intervals, and statistical significance was assessed using Wald or model-based *p* values. The forest plot depicts the point estimates and confidence intervals for each stage subgroup.

#### 2.8.5. Survival Analysis of Hub Genes Using cBioPortal

The cBioPortal for Cancer Genomics tool (https://www.cbioportal.org/ 4 April 2026) was used to evaluate the predictive value of identified hub genes. cBioPortal is an online facility that helps in easy access to comprehensive patient data from TCGA as well as publicly available data from cancer research studies [27]. Colorectal adenocarcinoma datasets containing survival information were selected. For each hub gene, patients were stratified into altered and unaltered groups according to genomic alteration status, where altered tumors included amplifications, mutations, or deep deletions. Kaplan–Meier analyses were performed for progression-free survival, disease-specific survival, disease-free survival, and overall survival, and differences between groups were assessed using the log-rank test with a two-sided alpha level of 0.05.

### 2.9. Machine Learning Assessment of the Gene Signature

An internal robustness assessment of the differential expression signature was performed using supervised machine learning in Google Colab with scikit-learn. To evaluate whether the 10-gene mitotic signature represents a reproducible expression pattern rather than random noise, we performed a limited machine learning analysis on the transcriptomic dataset. Standard supervised algorithms (linear regression, random forest, gradient boosting, support vector regression, and k-nearest neighbors) were evaluated for their ability to predict the degree of gene-expression dysregulation. This analysis was designed as an internal robustness and pattern-recognition check to evaluate the statistical consistency of the transcriptomic pattern within the studied datasets rather than to develop a clinically deployable prognostic model.

The data were randomly divided into training (70%) and test (30%) sets using a fixed random seed. The hub genes identified by the MCC method were included among the predictor features. Model performance was assessed using R^2^ and mean squared error (MSE). Missing values were removed when exceeding 20%, and remaining missing values were imputed using the mean. Five-fold cross-validation was applied during training to reduce overfitting. The hyperparameters of the gradient boosting regressor were optimized by grid search on the learning rate, the number of estimators, and the maximum depth.

## 3. Results

### 3.1. Data Normalization

After normalization, sample-level clustering showed improved comparability among datasets, supporting reliable downstream differential-expression analysis as shown in the box plots (Figure S1). The distribution of expression values across all samples within each group (tumor and normal) was normalized, with the median expression values indicating high data quality.

### 3.2. Identification of Differentially Expressed Genes

**Figure 1:**
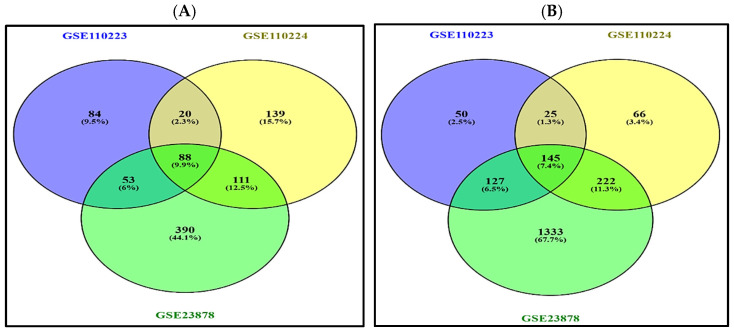
Comparison of Differentially Expressed Genes (DEGs) in Three Datasets (GSE110223, GSE110224, and GSE23878). (**A**) Upregulated genes; (**B**) Downregulated genes.

Differential expression analysis (DEA) was conducted on the selected datasets using the limma package in R, comparing CRC samples with normal tissue samples. The criteria for significance were adj *p* < 0.05 and |Log_2FC| > 1. Across the three combined datasets, a total of 2853 DEGs were identified. Among these, 885 genes were significantly upregulated, and 1968 were significantly downregulated. Additionally, 88 genes were commonly upregulated, and 145 genes were commonly downregulated across all three datasets (Figure 1). By integrating three independent GEO microarray datasets, we identified 88 commonly upregulated and 145 commonly downregulated genes across all cohorts, indicating reproducible transcriptional alterations in CRC. This cross-dataset consistency suggests that the signal is not attributable to dataset-specific noise but instead reflects a stable molecular feature of CRC biology.

**Figure 2:**
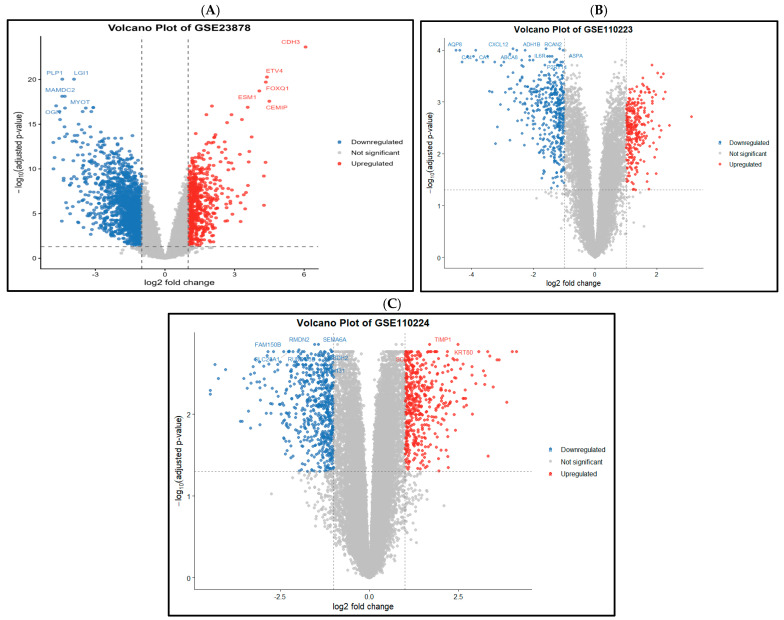
Volcano plots showing differential gene expression analysis comparing tumor versus normal samples in the datasets: (**A**) GSE23878, (**B**) GSE110223, and (**C**) GSE110224. Volcano plot of differential gene expression between colorectal cancer and matched normal tissue. Each point corresponds to a single gene. The *x*-axis shows log_2_ fold-change, where positive values indicate higher expression in cancer relative to normal tissue and negative values indicate higher expression in normal tissue relative to cancer. The *y*-axis shows −log_10_ of the adjusted *p*-value (FDR). Genes with FDR < 0.05 and an absolute log_2_ fold-change ≥ 1 (≥2fold change) are considered significantly differentially expressed. Significantly upregulated genes are highlighted in red (positive log_2_ fold-change), significantly downregulated genes in blue (negative log_2_ fold-change), and non-significant genes are shown in gray.

The DEA results are visualized in a volcano plot (Figure 2), which highlights the number and magnitude of statistically significant genes relative to the chosen thresholds.

Furthermore, the expression profiles of the top DEGs were visualized using hierarchical clustering and heatmap analysis (Figure S2). This approach demonstrated a clear separation and unsupervised clustering, confirming the molecular distinction between the tumor and normal sample cohorts.

### 3.3. Functional and Pathway Enrichment Analysis of DEGs

Functional annotation of the 233 Co-DEGs (88 upregulated, 145 downregulated) using Enrichr showed enrichment of biological process terms related to regulation of cell population proliferation (Figure S3). Molecular functions such as metallopeptidase activity and metal ion binding were also enriched (Figure S4).

The cellular components include the collagen-containing extracellular matrix (Figure S5). KEGG pathway analysis indicated significant enrichment of pathways related to rheumatoid arthritis and cell division (Figure S6).

### 3.4. Protein–Protein Interaction Network and Hub Gene Identification

**Figure 3:**
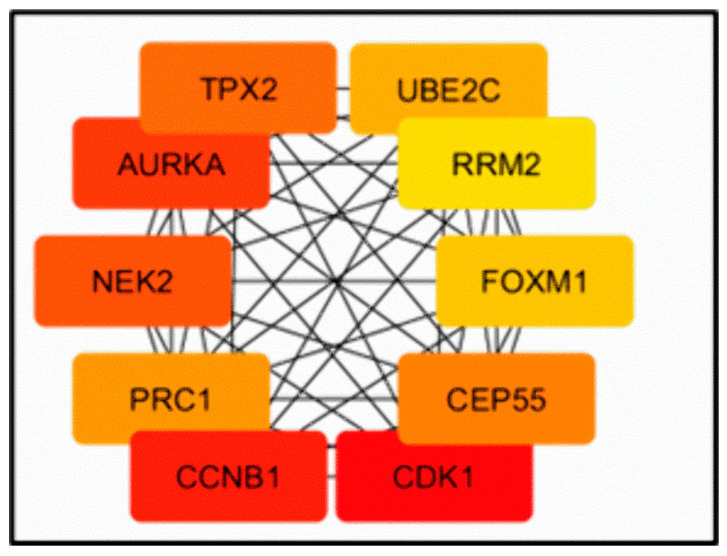
Hub gene Interaction Network. Each node represents a protein encoded by an input gene, and each edge indicates a known or predicted interaction supported by STRING evidence. Node color reflects maximal clique centrality (MCC), with more intense colors indicating nodes with higher MCC scores and therefore greater topological importance within the network. Edge thickness or presence reflects interaction confidence, where higher confidence scores indicate stronger support for the interaction. Only interactions above the selected confidence threshold were retained to reduce noise and improve interpretability.

The 233 Co-DEGs were submitted to the STRING database for the PPI network, resulting in a network composed of 186 nodes and 578 edges. Topological analysis with the MCC method in CytoHubba identified ten hub genes—*TPX2*, *UBE2C*, *AURKA*, *NEK2*, *PRC1*, *CCNB1*, *CDK1*, *CEP55*, *FOXM1*, and *RRM2*—all of which were significantly upregulated in CRC (Figure 3).

### 3.5. Protein Expression Assessment

**Figure 4:**
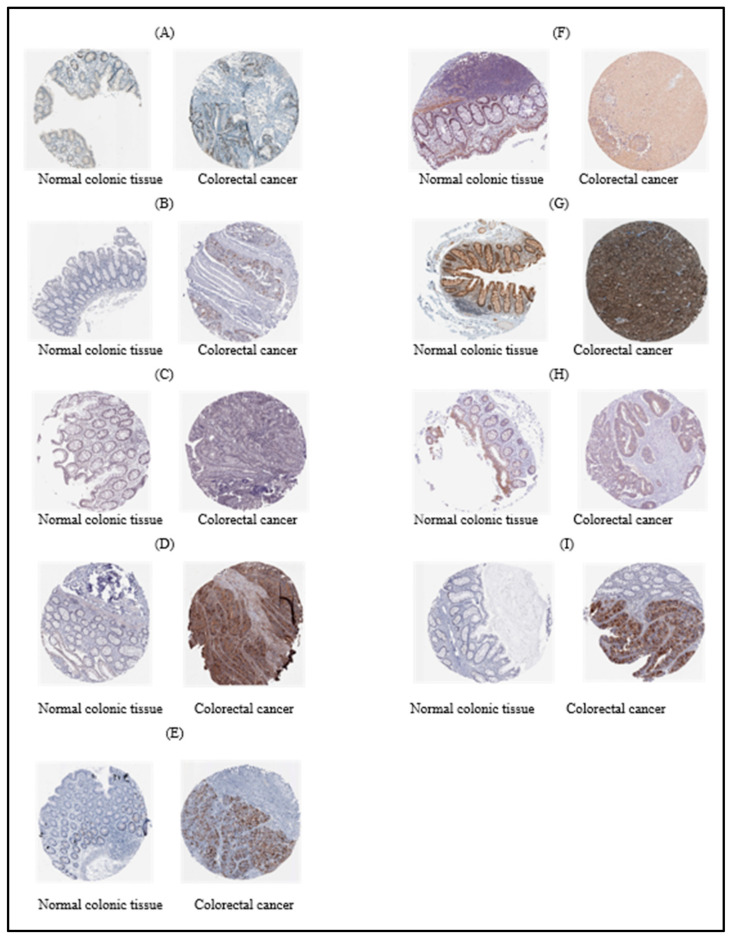
Representative IHC images obtained from the Human Protein Atlas showing expression of the hub proteins in normal colonic and colorectal tissues. (**A**) TPX2 staining by HPA005487 antibody, (**B**) AURKA staining by CAB001454 antibody, (**C**) NEK2 staining by CAB017530 antibody, (**D**) PRC1 staining by HPA034521 antibody, (**E**) CCNB1 staining by CAB000115 antibody, (**F**) CEP55 staining by HPA023430 antibody, (**G**) FOXM1 staining by CAB017832 antibody, (**H**) RRM2 staining by HPA056994 antibody, (**I**) UBE2C staining by CAB080407 antibody. Brown staining indicates positive immunoreactivity. The images are reproduced from HPA source material and interpreted with the corresponding source-provided scale information.

Immunohistochemical staining in the HPA showed increased protein expression of nine of the ten hub genes—TPX2, AURKA, NEK2, PRC1, CCNB1, CEP55, FOXM1, RRM2, and UBE2C—in CRC tissues compared with normal colonic tissues (Figure 4). NEK2 protein expression was not clearly detectable under the conditions evaluated.

### 3.6. Analysis of TCGA-COAD/READ Cohort

#### 3.6.1. Overall Survival by Mitotic Hub Gene Risk Group in the TCGA-COAD/READ Cohort

Overall survival did not differ significantly between patients classified into high and low mitotic hub gene risk groups in the full TCGA-COAD/READ cohort and stage II–III subset. In the Kaplan–Meier analysis, the survival curves for the two groups were largely overlapping, and the log-rank test yielded a non-significant log-rank *p* of 0.10, in the full TCGA-COAD/READ cohort (Figure S7A) and log-rank *p* of 0.34 in stage II–III subset (Figure S7B) indicating that dichotomization by the mitotic hub gene risk score alone did not provide meaningful stratification of overall mortality risk.

#### 3.6.2. Results of Multivariable Cox Models Analysis

Multivariable Cox models were fitted in both the full TCGA-COAD/READ cohort and the stage II–III subset. In the full TCGA-COAD/READ cohort, the mitotic hub gene risk score was not independently associated with overall survival (HR 0.85 per unit increase, 95% CI 0.65–1.12, *p* = 0.26), indicating no prognostic effect of the signature after accounting for age, sex, and stage. Age was entered as a large series of variables (age33–age90), which led to extreme hazard ratios with very large standard errors. This indicates that the individual age coefficients are numerically unstable and should not be interpreted clinically. Sex showed no significant association with survival (HR for males vs. females 1.01, 95% CI 0.63–1.62, *p* = 0.97), whereas advanced pathological stages remained strongly prognostic: stage IV and stage IVA were associated with markedly increased hazards of death compared with the reference (e.g., stage IV HR 7.10, 95% CI 4.08–12.36, *p* = 4.0 × 10^−12^; stage IVA HR 3.62, 95% CI 1.45–9.07, *p* = 0.006) (Table 1).

The global model performance measures suggested good overall discrimination (concordance index ≈ 0.84, likelihood ratio test *p* = 1 × 10^−4^, score test *p* = 5 × 10^−8^), driven primarily by stage and the overfitted age parameterization rather than by the mitotic hub gene risk score.

In the stage II–III TCGA-COAD/READ subset, the mitotic hub gene risk score showed only a borderline, non-significant association with overall survival (HR 0.73, 95% CI 0.51–1.05, *p* = 0.091), indicating that the signature did not provide clear independent prognostic information in stage II–III disease after adjustment for other factors. Male sex was significantly associated with worse overall survival compared with female sex (HR 2.91, 95% CI 1.52–5.56, *p* = 0.0013), suggesting that male patients with stage II–III colorectal cancer experienced approximately a three-fold higher risk of death. Stage contrasts within stage II–III showed that some categories were strongly associated with lower hazards relative to the reference stage (stage II: HR 0.11, 95% CI 0.04–0.28, *p* = 3.4 × 10^−6^, stage IIA: HR 0.27, 95% CI 0.14–0.52, *p* = 1.1 × 10^−4^, and stage IIIB: HR 0.22, 95% CI 0.09–0.53, *p* = 7.6 × 10^−4^) (Table 2). The global model performance metrics indicated high apparent discrimination (concordance index ≈ 0.90; likelihood ratio test *p* = 0.03; score test *p* = 3 × 10^−9^), reflecting the dominant impact of sex and pathological stage rather than a strong contribution from the mitotic hub gene risk score itself.

#### 3.6.3. Cox Model–Derived Prognostic Index and Tertile Risk Groups

**Figure 5:**
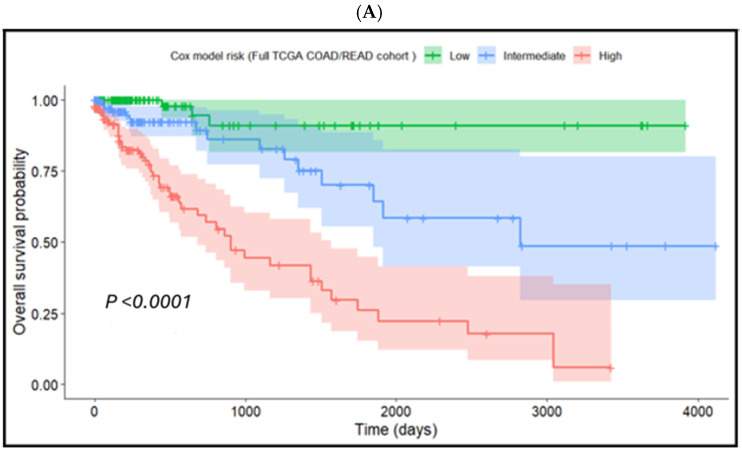

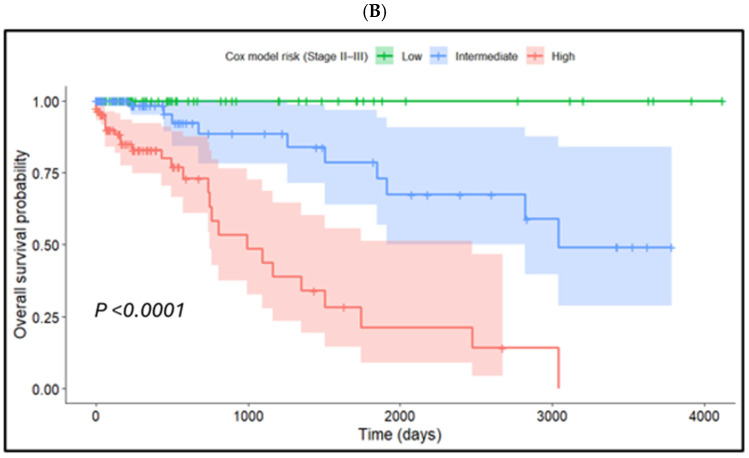
Overall survival by Cox model–derived tertiles. TCGA-COAD/READ cohort (**A**), stage II–III subset (**B**). Kaplan–Meier curves show overall survival for patients stratified into low-, intermediate-, and high-risk groups based on tertiles of the multivariable Cox model linear predictor, which incorporated the mitotic hub gene risk score, age, sex, and American Joint Committee on Cancer (AJCC) pathological stage; the curves show clear separation with the lowest mortality in the low-risk group and the highest in the high-risk group.

To assess whether the mitotic hub gene score could contribute to a broader integrated prognostic index, we derived a linear predictor from the multivariable Cox model of the TCGA-COAD/READ cohort and stage II–III subset (including mitotic hub score, age, sex, and stage) and stratified patients into tertiles of this Cox-derived risk score (low, intermediate, high). The Kaplan–Meier analysis of both the TCGA-COAD/READ cohort and the stage II–III subset showed significant stepwise separation of the three Cox risk groups (Figure 5A,B).

Patients in the low-risk tertile had the most favorable survival, with 1-, 3-, and 5-year overall survival rates of approximately 97%, 93%, and 93%, respectively, and median survival was not reached. The intermediate-risk group exhibited moderately reduced outcomes, with 1-, 3-, and 5-year survival of roughly 90%, 84%, and 68%, and an estimated median survival around 2821 days (~7.7 years; 95% CI 2134–NA). The high-risk group had the poorest outcomes, with 1-, 3-, and 5-year survival of about 79%, 56%, and 40%, and a median survival of ~1331 days (~3.6 years; 95% CI 1094–2003) (Table 3). The global log-rank test was highly significant (χ^2^ ≈ 60.8, df = 2, *p* ≈ 6 × 10^−14^), with substantially fewer deaths than expected in the low-risk group and far more deaths than expected in the high-risk group.

In the stage II–III subset, patients in the low-risk tertile had excellent outcomes, with median overall survival not reached and estimated 1-, 3-, and 5-year survival probabilities of 1.00, 1.00, and 1.00, respectively. The intermediate-risk tertile showed moderately reduced survival, with a median overall survival of 3042 days (95% CI 2821–NA) and 1-, 3-, and 5-year survival probabilities of 0.98, 0.89, and 0.79, respectively. In contrast, the high-risk tertile experienced substantially poorer outcomes, with a median overall survival of 992 days (95% CI 743–2475) and corresponding 1-, 3-, and 5-year survival probabilities of 0.83, 0.44, and 0.21 (Table 4). These findings indicate that, although the mitotic hub gene risk score alone was modest, integrating it with clinical covariates into a Cox-derived prognostic index allowed effective risk stratification of stage II–III patients into distinct survival groups.

#### 3.6.4. Time-Dependent ROC

**Figure 6:**
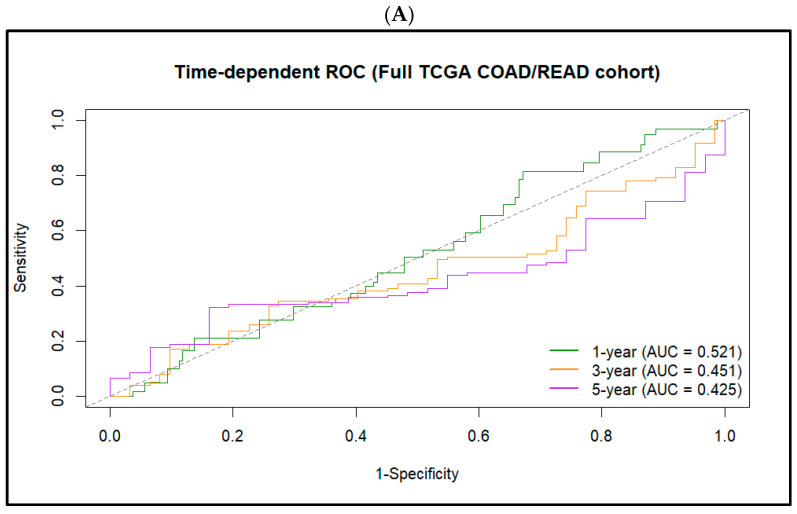

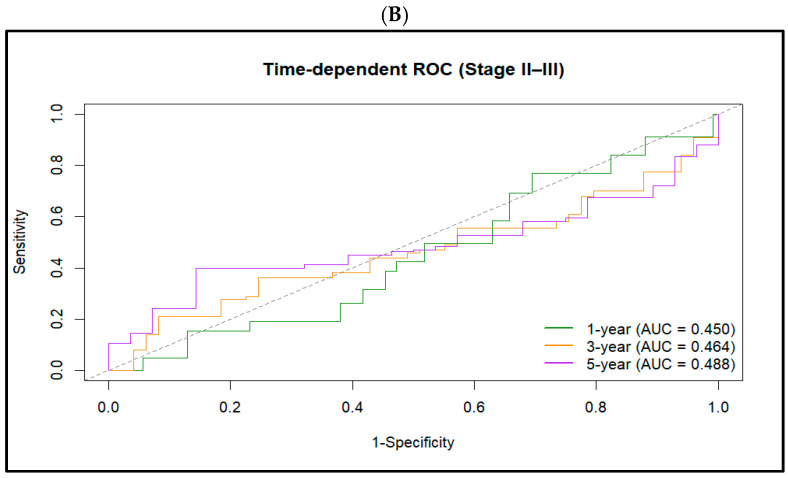
Time-dependent ROC curves of the mitotic hub gene signature for overall survival in the TCGA-COAD/READ cohort (**A**) and stage II–III subset (**B**). Time-dependent receiver operating characteristic (ROC) curves evaluating the prognostic performance of the ten-gene mitotic hub signature for overall survival in the TCGA-COAD/READ cohort and stage II–III subset. The curves depict sensitivity versus 1 − specificity for predicting 1-year (green), 3-year (orange), and 5-year (purple) overall survival based on the continuous signature-derived risk score. The diagonal dashed line represents the performance of a non-informative classifier (AUC = 0.5).

Time-dependent ROC analysis for the continuous mitotic hub risk score in the full TCGA-COAD/READ cohort demonstrated weak discrimination over time. The AUCs at 1, 3, and 5 years were 0.52, 0.45, and 0.43, respectively, and the corresponding 95% confidence intervals all encompassed 0.5 (Figure 6A). This indicates that the signature alone did not materially outperform chance for the prediction of short-, intermediate-, or longer-term overall survival. In the stage II–III TCGA subset, the time-dependent ROC curves for the continuous mitotic hub gene risk score showed modest and statistically uncertain discrimination for overall survival. The AUCs at 1, 3, and 5 years were approximately 0.45, 0.46, and 0.49, respectively, with 95% confidence intervals spanning 0.5 at all-time points (1-year AUC 95% CI 0.29–0.61; 3-year AUC 95% CI 0.29–0.63; 5-year AUC 95% CI 0.31–0.66 (Figure 6B). These findings indicate that in stage II–III disease, the mitotic hub gene signature provides at best weak and imprecise incremental prognostic information over a 1–5-year horizon.

#### 3.6.5. Survival Stratification by PCA-Derived Signature Score

**Figure 7:**
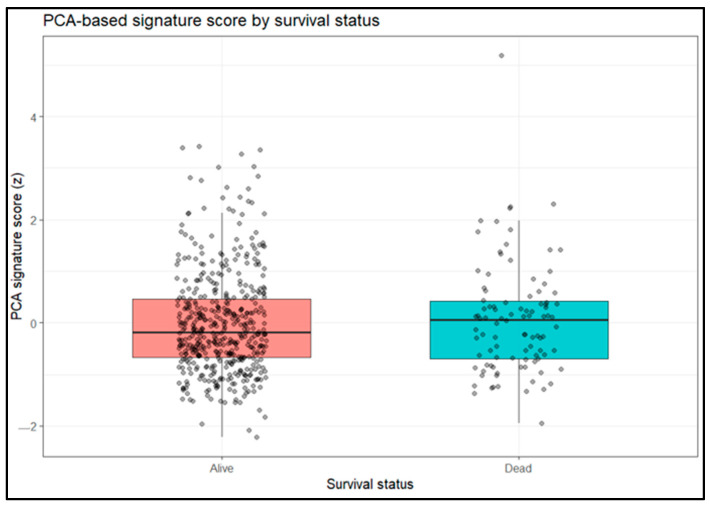
PCA-based signature score by survival status.

The PCA-derived signature score showed similar distributions in patients who were alive versus those who had died at last follow-up. The box-and-jitter plot illustrates considerable overlap in the median and interquartile ranges of signature_score_pca_z between the two groups (Figure 7). This pattern suggests that, in this cohort, the PCA-based 10-gene mitotic signature does not exhibit a strong univariate separation between survival-status groups.

Kaplan–Meier analysis showed that the high PCA signature score group had a trend toward worse overall survival than the low score group, although this difference was not statistically significant (*p* = 0.34) (Figure S8). The survival curves separated gradually over follow-up, with the high-score group showing lower survival probability at later time points.

#### 3.6.6. Stage Distribution and Cox Regression Analysis of the PCA-Based Signature

**Figure 8:**
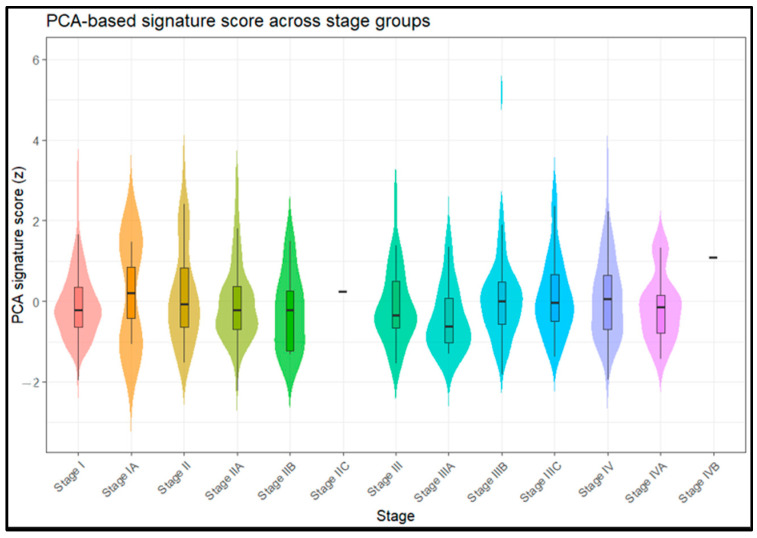
PCA-based signature score across stage groups.

The PCA-based signature score varied across the pathologic stage categories, with the violin plots indicating subtle differences in score distributions between early and advanced stages (Figure 8). While the overall spread of signature_score_pca_z was wide within most stages; some higher stages (Stage III, IIIC, IV, IVA) appeared to have slightly elevated median scores or longer upper tails compared with lower stages, consistent with modest enrichment of the mitotic signature in more advanced disease. However, the substantial overlap between stage distributions indicates that the PCA-derived score captures a continuous gradient rather than sharply separating discrete stage groups.

**Figure 9:**
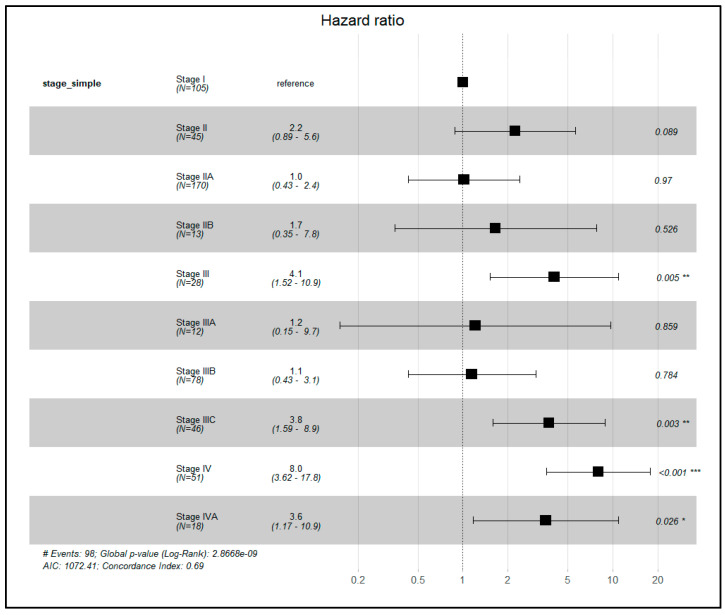
Forest plot of hazard ratios for stage categories relative to stage I. * *p* < 0.05, ** *p* < 0.01, *** *p* < 0.001.

In the Cox model, higher disease stage was associated with increased mortality risk compared with stage I. Significant associations were observed for stage III (HR 4.1, 95% CI 1.52–10.9, *p* = 0.005), stage IIIC (HR 3.8, 95% CI 1.59–8.9, *p* = 0.003), stage IV (HR 8.0, 95% CI 3.62–17.8, *p* < 0.001), and stage IVA (HR 3.6, 95% CI 1.17–10.9, *p* = 0.026) (Figure 9). Other stage subgroups were not statistically significant. The model showed good prognostic performance overall, with a concordance index of 0.69 and a global log-rank *p* value of 2.8668 × 10^−9^.

### 3.7. Survival and Clinical Relevance Analysis

The prognostic value of the 10 hub genes was evaluated using the TCGA Colorectal Adenocarcinoma dataset through the cBioPortal tool. Our result revealed that genetic alterations in the identified hub genes were significantly associated with adverse survival outcomes.

#### 3.7.1. Disease-Specific Survival

Kaplan–Meier analysis showed alterations in *TPX2* were significantly associated with worse disease-specific survival (DSS), with a log-rank *p* value of 0.0152 (Figure S9A), indicating that *TPX2* status is an important prognostic factor for disease-specific mortality in CRC. Similarly, patients with alterations in PRC1 had significantly reduced DSS compared with the unaltered group (log-rank *p* = 0.0193) (Figure S9B), underscoring the prognostic relevance of *PRC1* as a potential biomarker.

#### 3.7.2. Progression-Free Survival

Patients harboring genetic alterations in *TPX2* showed significantly reduced progression-free survival (PFS) (*p* value = 3.335 × 10^−3^) compared with patients without *TPX2* alterations (Figure S10).

#### 3.7.3. Disease-Free Survival

Alterations in the *RRM2* gene were found to be significantly associated with reduced disease-free survival (DFS), as patients with *RRM2* alterations exhibited shorter DFS than those without alterations (log-rank *p* = 0.0212) (Figure S11).

#### 3.7.4. Overall Survival

Patients harboring *FOXM1* alterations showed markedly reduced overall survival compared with those without alterations (log-rank *p* = 0.0140) (Figure S12). This significant difference indicates that *FOXM1* alterations confer an adverse impact on patient prognosis and support *FOXM1* as a potential unfavorable prognostic biomarker.

### 3.8. Higher Mitotic Hub Scores in TCGA-COAD/READ Tumor than Normal Samples

**Figure 10:**
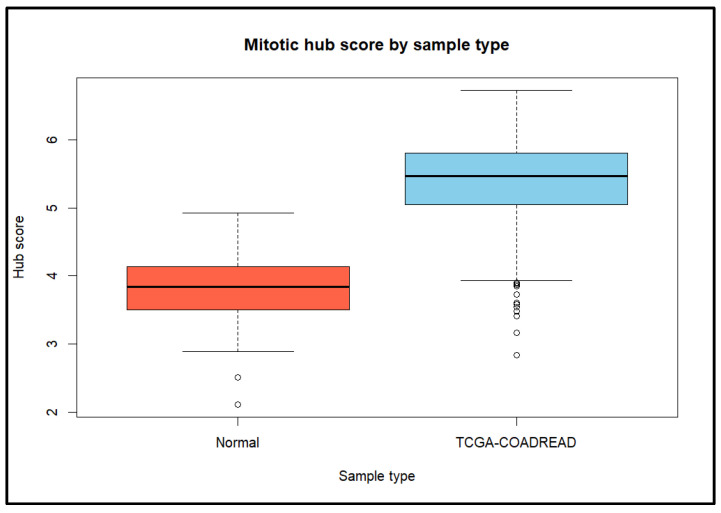
Mitotic hub score of TCGA-COAD/READ Tumor and normal samples.

The mitotic hub score was markedly higher in TCGA-COAD/READ samples than in normal samples. The boxplot showed a clear upward shift in hub score in the tumor group (Figure 10).

This difference was highly significant by Wilcoxon rank-sum test (W = 1052, *p* < 2 × 10^−16^) and was also confirmed by one-way ANOVA (F = 336.52, *p* < 2 × 10^−16^), indicating a strong association between the ten-gene mitotic signature and tumor tissue.

### 3.9. Mitotic Hub Score Strongly Correlates with the Proliferation Score in TCGA-COAD/READ

**Figure 11:**
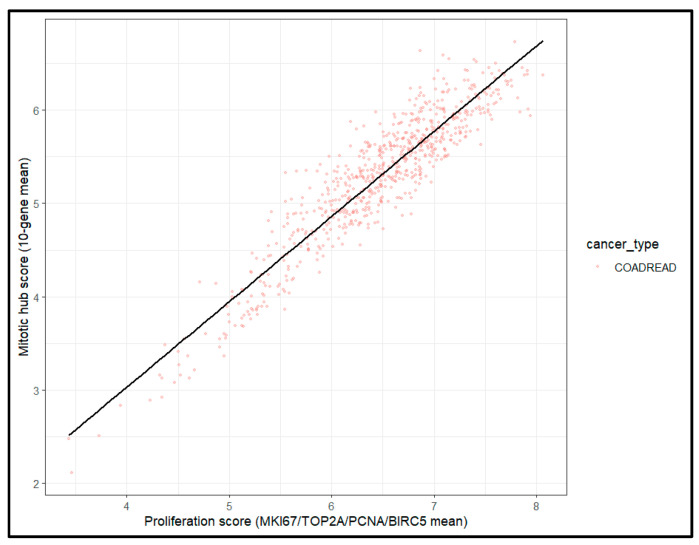
Scatterplot of mitotic hub score versus proliferation score in TCGA-COAD/READ samples. Each point represents one sample, and the fitted line indicates a strong positive association between the two scores.

In the TCGA-COAD/READ cohort, the mitotic hub score showed a strong positive correlation with the proliferation score. Among 701 samples, the Pearson correlation was 0.926, and the Spearman correlation was 0.898, indicating that the ten-gene hub signature closely tracks a general proliferative transcriptional program (Figure 11). Because only COADREAD was available in the current analysis, this result reflects a single cancer type rather than a true multi-cancer comparison.

### 3.10. Machine Learning–Based Robustness Assessment

Among the evaluated regression models, the Gradient Boosting Regressor achieved the best performance, with an R^2^ of 0.8035 and an MSE of 0.0473 on the test set (Table 5).

**Figure 12:**
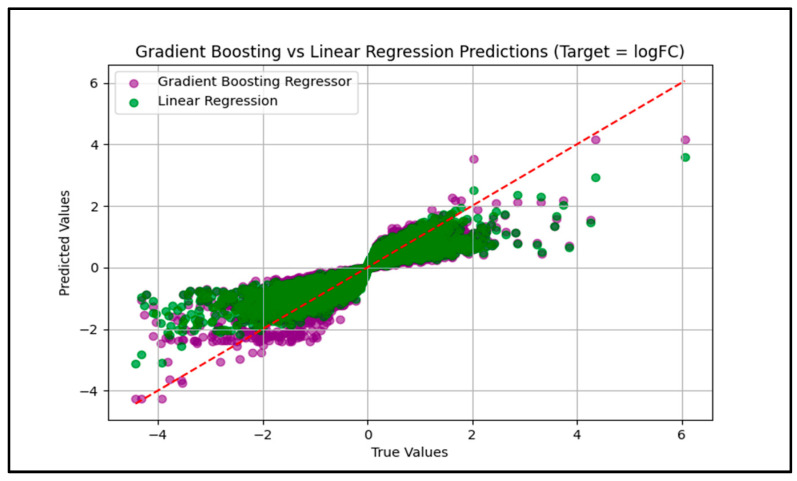
Scatterplot of True vs. Predicted log_2_ FC Values. Gradient Boosting vs. Linear Regression.

Figure 12 presents a comparison between the Gradient Boosting algorithm and linear regression in predicting log_2_FC values. Both models showed a positive relationship between predicted values on the *y*-axis and actual values on the *x*-axis. However, the predictions from the Gradient Boosting regression model tended to lie closer to the ideal line, indicating better agreement with the true values. In contrast, predictions from the linear regression model showed greater deviation, particularly at the extremes. This figure illustrates that the Gradient Boosting regression model is more accurate than linear regression in predicting log_2_FC.

These machine learning models were able to learn the mitotic expression pattern from the high-dimensional data, supporting that the 10-gene signature corresponds to a stable, reproducible proliferative program in CRC rather than idiosyncratic noise. Importantly, these findings are exploratory and do not by themselves demonstrate disease specificity or independent prognostic utility.

## 4. Discussion

A ten-gene mitotic hub signature, comprising TPX2, UBE2C, AURKA, NEK2, PRC1, CCNB1, CDK1, CEP55, FOXM1, and RRM2, was identified in colorectal cancer through an integrated workflow combining transcriptomic analysis, network topology, protein-level assessment, survival analysis, and machine learning evaluation. Overall, the findings support the biological relevance of mitotic dysregulation in CRC and indicate that the signature reflects a coordinated proliferative program associated with tumor progression rather than isolated transcriptional changes. At the same time, the results indicate that the signature has limited value as a standalone prognostic marker. This interpretation is supported by the tight functional connectivity observed in the PPI network, as well as by protein-level evidence from public immunohistochemistry resources showing generally increased expression in CRC tissues. Several of these genes have also been implicated previously in colorectal tumor progression and mitotic control, reinforcing the biological plausibility of our findings. PRC1, CCNB1, CEP55, FOXM1, RRM2, and UBE2C are known to participate in cytokinesis, cell-cycle regulation, and DNA synthesis [28,29,30,31,32,33,34,35]. The absence of clearly detectable NEK2 protein expression highlights the importance of post-transcriptional regulation, protein stability, and assay-specific factors in biomarker interpretation. This further emphasizes that transcriptomic findings do not necessarily translate directly into protein abundance and therefore require independent validation [36,37].

An important interpretive issue is that the discovery datasets were bulk tissue transcriptomes rather than microdissected tumor cell profiles. Consequently, part of the observed differential expression may reflect differences in stromal, immune, vascular, or inflammatory composition between tumor and normal tissues rather than tumor cell–intrinsic biology alone. This limitation is particularly relevant for enrichment of extracellular matrix and inflammatory pathways, which may partly reflect microenvironment remodeling. Therefore, the findings should be interpreted as tissue-level tumor-associated transcriptional programs rather than purely epithelial-cell signatures. In this sense, the mitotic hub signature likely captures a combination of tumor proliferation and microenvironment-associated remodeling, and future studies will be needed to separate these components more definitively.

The prognostic analyses showed that the mitotic signature had limited value as a standalone survival marker. In the TCGA-COAD/READ cohort, the signature did not provide strong separation of survival groups, was not independently associated with overall survival, and showed weak time-dependent discrimination. Notably, the signature became more informative when incorporated into a multivariable clinical–genomic model, suggesting that its main value may lie in adding biological context to established clinicopathologic variables rather than replacing them. However, these findings should be interpreted cautiously because the improved performance in the multivariable setting largely reflects the contribution of established clinical variables, especially pathological stage.

Although the PCA-derived signature did not significantly improve prognostic prediction beyond the stage in the full cohort, this does not exclude its potential value as a complementary marker. Stage is a strong and expected predictor of survival, and the more relevant question for a molecular signature is whether it can refine risk within stage-defined groups. In the present study, the signature showed biologic coherence and some association with stage, but additional stage-stratified analyses will be required to determine whether it can meaningfully improve risk discrimination in specific clinical subgroups. Therefore, the current findings support the signature as a biologically informative marker of proliferation, but not yet as an independently validated prognostic tool beyond standard staging.

The significantly higher mitotic hub score in TCGA-COAD/READ tumor samples and its strong correlation with a proliferation score suggest that the ten-gene signature reflects a tumor-associated proliferative program in colorectal cancer. These findings support the biological relevance of the signature and are consistent with the known roles of the included genes in mitosis and cell-cycle control. However, because the analysis was limited to COADREAD and did not include formal pan-cancer or subtype-specific comparison, these results do not establish CRC specificity. Therefore, the signature should be interpreted as a CRC-associated mitotic axis with clear biological coherence but unproven disease specificity. This result strengthens the mechanistic interpretation of the signature and supports its use as a marker of mitotic activity rather than as a stand-alone prognostic biomarker. In the broader context of the study, the result helps explain why the signature showed strong tumor-associated expression differences but limited independent survival prediction, suggesting that its main value lies in reflecting CRC biology and contributing to integrated clinical–genomic interpretation.

The machine learning analysis was exploratory and intentionally restricted to an internal robustness assessment of the mitotic signature rather than clinical prognostic utility. Our results showed that standard algorithms can recover the signature-derived signal from the full transcriptome; these analyses support the idea that the 10-gene set captures a coherent proliferative pattern. However, they do not establish disease specificity or incremental prognostic value and should be viewed as exploratory pattern recognition rather than a clinically validated prediction tool. In this context, the machine learning results should be viewed as methodological support for the coherence of the signature rather than as validation of prognostic utility.

The main novelty of this study is not the construction of a prognostic score but the demonstration that a reproducible ten-gene mitotic program can be identified across datasets, supported at the protein level, and quantitatively linked to proliferation and tumor state while showing limited independent prognostic power beyond stage. Future work will need to extend this framework to independent cohorts, clinically relevant end points, and formally defined predictive tasks.

A major strength of this study is its multi-layer design, which integrates transcriptomic discovery, network-based prioritization, protein-level assessment, gene-level survival analysis, multivariable modeling, and machine learning-based robustness assessment. Although the analytical workflow employed here is standard in bioinformatics studies, the convergent signal observed across independent datasets supports the reproducibility of the identified mitotic expression program in colorectal cancer. This framework allows biological interpretation to be separated from prognostic modeling and provides a more balanced assessment than studies that rely solely on differential expressions or single-cohort survival associations. Another strength is the explicit reporting of negative findings, particularly the limited standalone prognostic performance of the mitotic hub score, which helps avoid overstating clinical relevance.

The present study should be viewed as an exploratory characterization of a mitotic hub-gene program in colorectal cancer. Although the computational workflow is conventional and similar integrative pipelines are widely used in bioinformatics studies, the reproducible convergence of differential expressions, network topology, protein-level patterns, and survival associations across independent datasets supports the robustness of the identified biological signal. Nevertheless, further validation in independent cohorts and experimental systems will be required to determine its specificity and translational relevance.

### 4.1. Limitations

This study has several limitations that should be considered when interpreting the findings. First, the analysis relied on publicly available retrospective transcriptomic datasets generated on different Affymetrix platforms, which may introduce residual technical and batch-related variation despite within-dataset normalization. Second, we did not perform formal tumor purity correction, stromal or immune deconvolution, or other microenvironment adjustment analyses; therefore, the observed signal may partially reflect differences in tumor composition rather than cancer-cell intrinsic biology. Third, the identified mitotic hub signature was not validated in independent external cohorts, nor was it confirmed experimentally by qRT-PCR, immunoblotting, immunohistochemistry, or functional assays. Fourth, the machine learning analysis was used only as an internal robustness check and was not designed to establish a clinically deployable predictive model. Finally, although the signature was associated with altered expression cancers. Definitive assessment of specificity will require a true pan-cancer tumor-only comparison and subtype-level analyses across additional TCGA cohorts, which are beyond the scope of the current revision.

### 4.2. Future Directions

Future studies should address these limitations by validating the ten-gene signature in independent CRC cohorts, preferably using harmonized transcriptomic platforms and prospectively collected clinical samples. Experimental confirmation in tissue-based and functional systems will also be important to verify the biological relevance of the candidate hub genes. In addition, formal assessment of batch effects, tumor purity, stromal composition, and immune infiltration should be incorporated to determine whether the signal remains robust after adjustment for microenvironmental confounding. Pan-cancer comparison, microsatellite instability (MSI), chromosomal instability (CIN) status, and consensus molecular subtypes (CMS) annotation would help determine whether the signature is truly CRC-specific or represents a generic mitotic/proliferative program. Finally, any future prognostic model should evaluate whether the signature adds incremental value beyond established clinicopathologic variables such as stage, rather than being judged in isolation.

### 4.3. Conclusions

In this CRC cohort, the PCA-derived 10-gene mitotic signature is a biologically coherent marker of proliferation in the observed CRC cohort, shows modest associations with stage and survival, and does not add substantial prognostic value beyond standard staging. The broader clinical utility requires validation in independent, well-characterized datasets.

Taken together, these findings should be interpreted as an exploratory, hypothesis-generating characterization of a CRC-associated mitotic program rather than as a fully validated mechanistic or clinically deployable model. Further validation in independent cohorts and experimental systems will be required to determine its disease specificity and translational value.

## Figures and Tables

**Table 1:** Multivariable Cox analysis of the mitotic hub gene signature and clinicopathologic factors in the full TCGA-COAD/READ cohort.

Variable	HR	95% CI	*p*-Value
Mitotic hub gene risk score	0.85	0.65–1.12	0.26
Sex: male vs. female	1.01	0.63–1.62	0.97
Stage [Not available] vs. ref	3.53	1.39–8.96	0.008
Stage I vs. reference	0.40	0.16–1.01	0.052
Stage II vs. reference	0.63	0.26–1.52	0.31
Stage IIA vs. reference	0.44	0.24–0.81	0.008
Stage IIB vs. reference	0.78	0.11–5.68	0.81
Stage III vs. reference	0.41	0.05–3.13	0.39
Stage IIIA vs. reference	2.33	0.57–9.60	0.24
Stage IIIB vs. reference	0.61	0.26–1.42	0.25
Stage IIIC vs. reference	1.21	0.60–2.44	0.60
Stage IV vs. reference	7.10	4.08–12.36	3.96 × 10^−12^
Stage IVA vs. reference	3.62	1.45–9.07	0.006

Global model performance: concordance index 0.84 (SE 0.024); likelihood ratio test *p* = 1 × 10^−4^; score test *p* = 5 × 10^−8^.

**Table 2:** Multivariable Cox analysis of the mitotic hub gene signature and clinicopathologic factors in the stage II–III subset of the TCGA-COAD/READ cohort.

Variable	HR	95% CI	*p*-Value
Mitotic hub gene risk score	0.73	0.51–1.05	0.091
Sex: male vs. female	2.91	1.52–5.56	0.0013
Stage II vs. reference	0.11	0.04–0.28	3.4 × 10^−6^
Stage IIA vs. reference	0.27	0.14–0.52	1.1 × 10^−4^
Stage IIB vs. reference	0.38	0.05–2.79	0.34
Stage IIC vs. reference	~0.00	0–∞	0.99
Stage III vs. reference	0.33	0.04–2.56	0.29
Stage IIIA vs. reference	0.69	0.16–2.98	0.62
Stage IIIB vs. reference	0.22	0.09–0.53	7.6 × 10^−4^
Stage IIIC vs. reference	1.00	0.46–2.18	1.00

Global model performance: concordance index ≈ 0.90; likelihood ratio test *p* = 0.03; score test *p* = 3 × 10^−9^.

**Table 3:** Survival probabilities for 1-year, 3-year, and 5-year overall survival (OS) of the TCGA-COAD/READ cohort.

Risk Group	Median OS (Days)	95% CI (Days)	1-Year OS	3-Year OS	5-Year OS
Low	Not reached	–	0.97	0.93	0.93
Intermediate	2821	2134–NA	0.90	0.84	0.68
High	1331	1094–2003	0.79	0.56	0.40

**Table 4:** Survival probabilities for 1-year, 3-year, and 5-year overall survival (OS) of the stage II–III subset.

Cox Risk Group	Median OS (Days, 95% CI)	1-Year OS	3-Year OS	5-Year OS
Low	Not reached	1.00	1.00	1.00
Intermediate	3042 (2821–NA)	0.98	0.89	0.79
High	992 (743–2475)	0.83	0.44	0.21

**Table 5:** Comparison of Regression Model Performance.

Model	MSE	R^2^
Linear Regression	0.0517	0.7851
Random Forest	0.0623	0.7408
Decision Tree	0.0860	0.6425
Gradient Boosting	0.0473	0.8035
Support Vector Regressor	0.0516	0.7856
KNN Regressor	0.0597	0.7518

MSE: Mean Squared Error; R^2^ Coefficient of determination Score.

## Data Availability

The datasets supporting the conclusions of this article are available in the Zenodo repository at https://doi.org/10.5281/zenodo.20927885.

## Supplementary Materials

The following supporting information can be downloaded at: https://www.mdpi.com/article/10.3390/genes17070783/s1.
