# Supplementary material for: Mitotic Hub Gene Network in Colorectal Cancer: Integrated Transcriptomic, Protein-Level, and Clinical-Genomic Characterization of a Ten-Gene Signature
Source: Genes (Basel). 2026 Jul 8;17(7):783. doi: 10.3390/genes17070783 (PMC13408991; doi:10.3390/genes17070783)
Supplement: Supplementary file 1 [file genes-17-00783-s001.zip › genes-4392595-supplementary.pdf]

Table S1. Description of the GEO datasets.

Accession Num	Sample Numbers / Characteristics	Platform Informatio	Reference
GSE110223	Paired human colorectal adenocarcinoma and adjacent non-cancerous tissues (n=13)	Affymetrix GPL96	Linked to PMID: 30809322
GSE110224	Paired human colorectal adenocarcinoma tissues and adjacent non-cancerous tissues (n=17)	Affymetrix GPL570	Linked to PMID: 30809322
GSE23878	35 colorectal cancer samples versus 24 normal samples	Affymetrix GPL570	Linked to PMID: 21281787

(A)

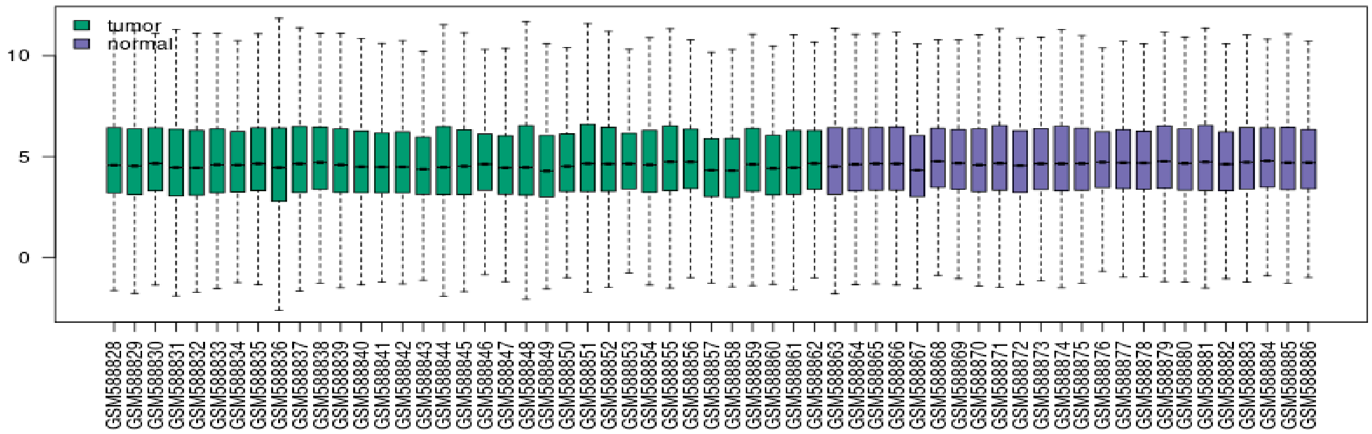

(B)

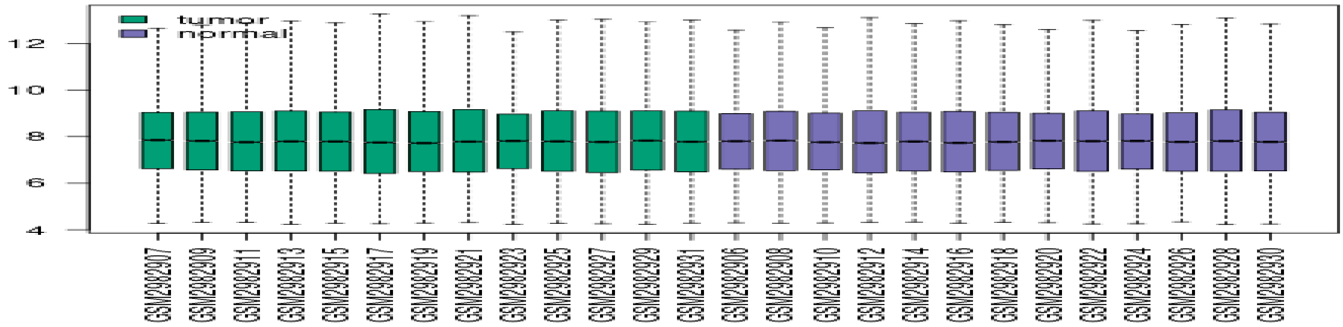

(C)

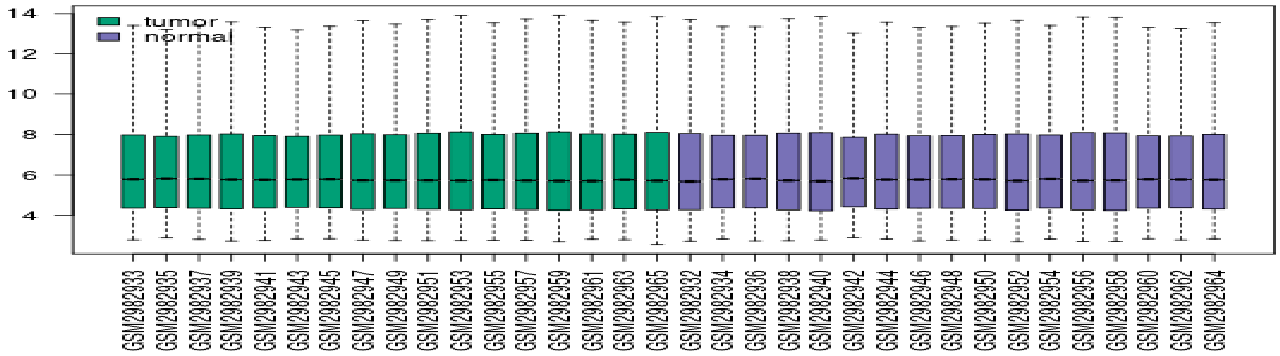

Figure S1. Box plot showing the distribution of gene expression in tumor and normal samples for the dataset: (A) GSE23878, (B) GSE110223, and (C) GSE110224.

(A)

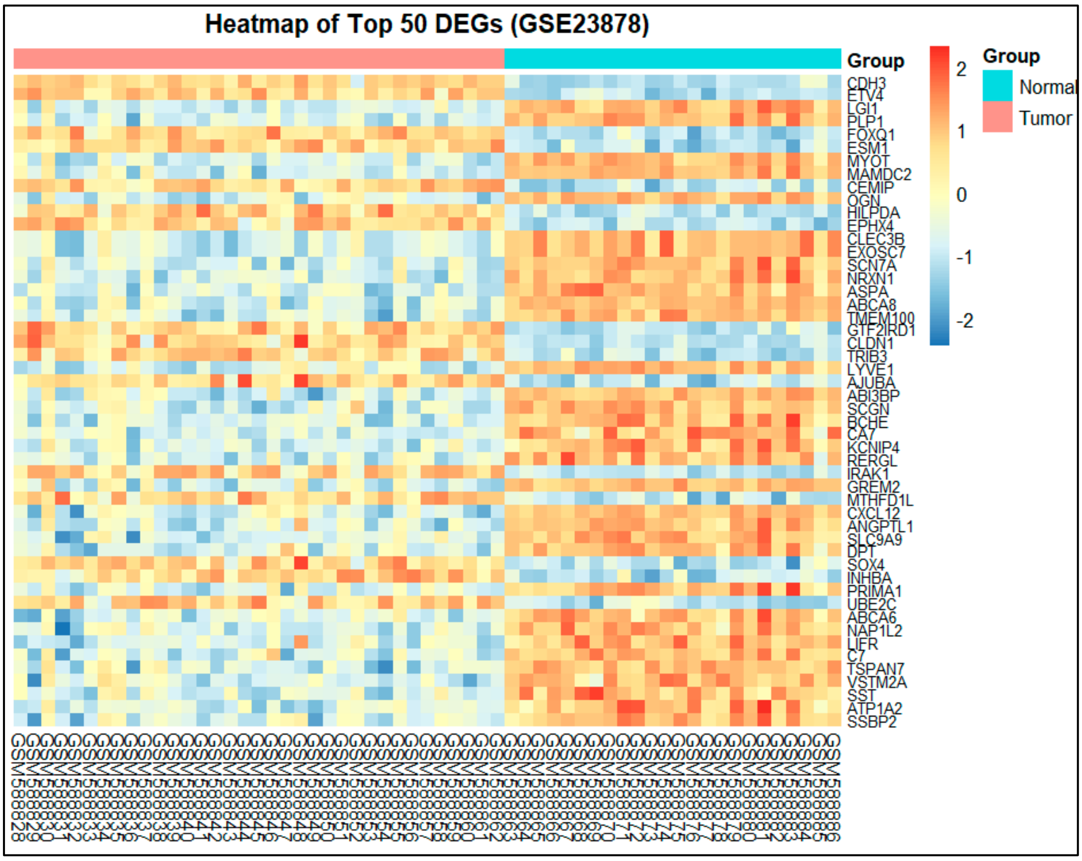
Heatmap of Top 50 DEGs (GSE23878)
Group
Group
Normal
Tumor
2
1
0
-1
-2
CDH3
ETV4
LGI1
PLP1
FOXQ1
ESM1
MYOT
MAMDC2
CEMIP
OGN
HILPDA
EPHX4
CLEC3B
EXOSC7
SCN7A
NRXN1
ASPA
ABCA8
TMEM100
GTF2IRD1
CLDN1
TRIB3
LYVE1
AJUBA
ABI3BP
SCGN
BCHE
CA7
KCNIP4
RERGL
IRAK1
GREM2
MTHFD1L
CXCL12
ANGPTL1
SLC9A9
DPT
SOX4
INHBA
PRIMA1
UBE2C
ABCA6
NAP1L2
LIFR
C7
TSPAN7
VSTM2A
SST
ATP1A2
SSBP2

(B)

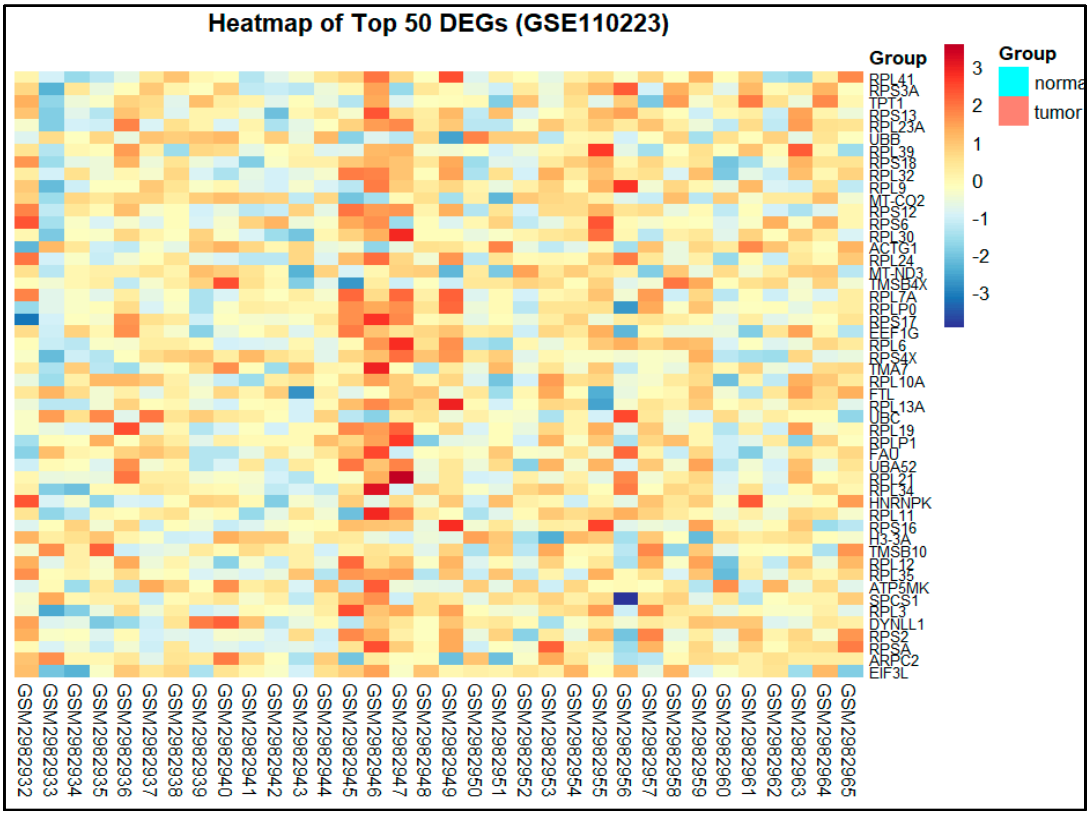
Heatmap of Top 50 DEGs (GSE110223)
Group
Group
norma
tumor
3
2
1
0
-1
-2
-3
RPL41
RPS3A
TPT1
RPS13
RPL23A
UBB
RPL39
RPS18
RPL32
RPL9
MT-CO2
RPS12
RPS6
RPL30
ACTG1
RPL24
MT-ND3
TMSB4X
RPL7A
RPLP0
RPS17
EEF1G
RPL6
RPS4X
TMA7
RPL10A
FTL
RPL13A
UBC
RPL19
RPLP1
FAU
UBA52
RPL21
RPL34
HNRNPK
RPL11
RPS16
H3-3A
TMSB10
RPL12
RPL35
ATP5MK
SPCS1
RPL3
DYNLL1
RPS2
RPSA
ARPC2
EIF3L
GSM2982932
GSM2982933
GSM2982934
GSM2982935
GSM2982936
GSM2982937
GSM2982938
GSM2982939
GSM2982940
GSM2982941
GSM2982942
GSM2982943
GSM2982944
GSM2982945
GSM2982946
GSM2982947
GSM2982948
GSM2982949
GSM2982950
GSM2982951
GSM2982952
GSM2982953
GSM2982954
GSM2982955
GSM2982956
GSM2982957
GSM2982958
GSM2982959
GSM2982960
GSM2982961
GSM2982962
GSM2982963
GSM2982964
GSM2982965

(C)

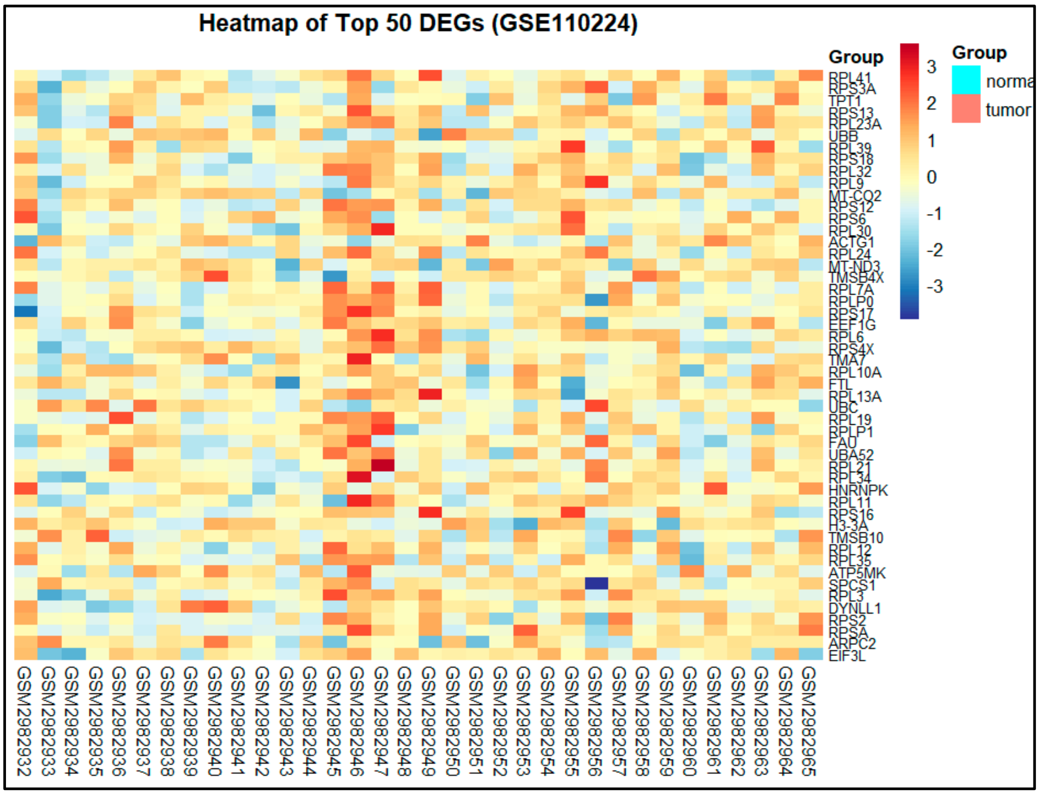

Figure S2. Heatmap of the top differentially expressed genes across datasets GSE23878 (A), GSE110223 (B), and GSE110224 (C).

Each column represents one sample and each row one gene. Expression values are centered and scaled per gene. Fold changes are shown as log2 values, where positive values indicate upregulation in cancer relative to normal tissue and negative values indicate downregulation in cancer. The diverging blue white–red colour scale encodes log2 fold change, with blue denoting decreased expression, red denoting increased expression, and white indicating no change. Genes were selected based on an adjusted p-value (FDR) < 0.05 and an absolute log2 fold-change ≥ 1

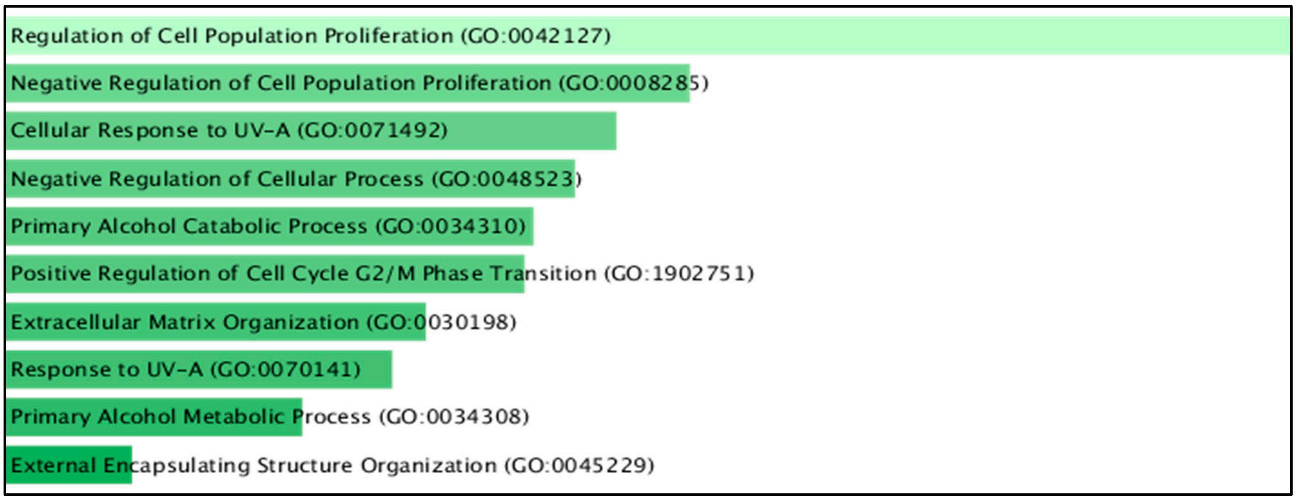

Figure S3. Biological processes associated with the differentially expressed genes.

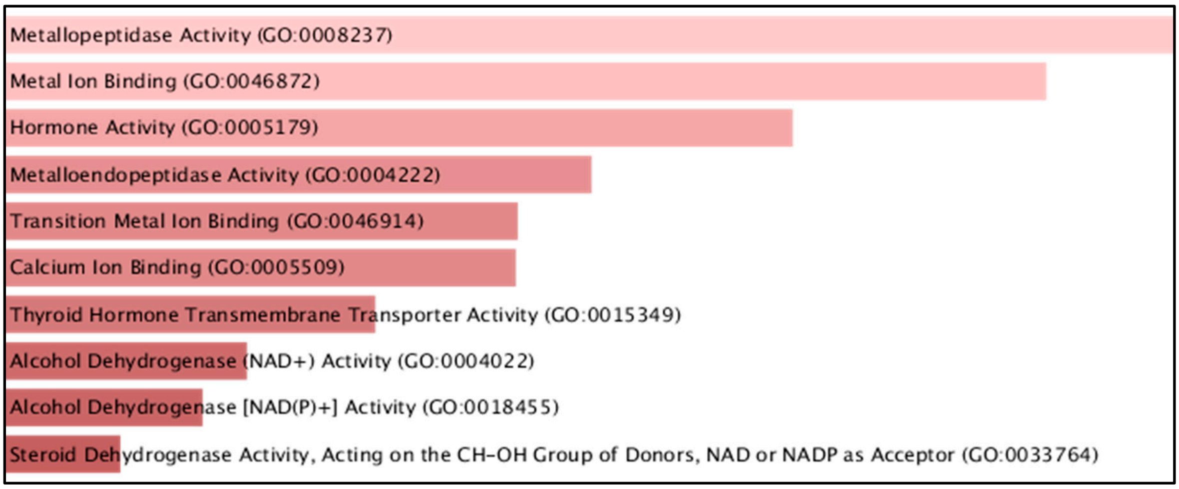

Figure S4. Molecular Functions of the differentially expressed gene.

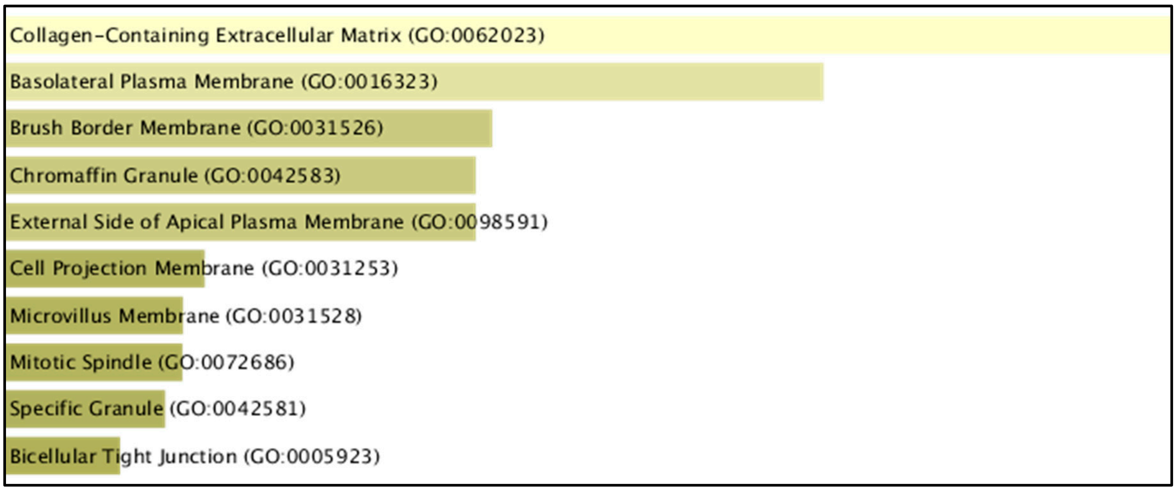

Figure S5. Cellular components of the Differentially Expressed Genes.

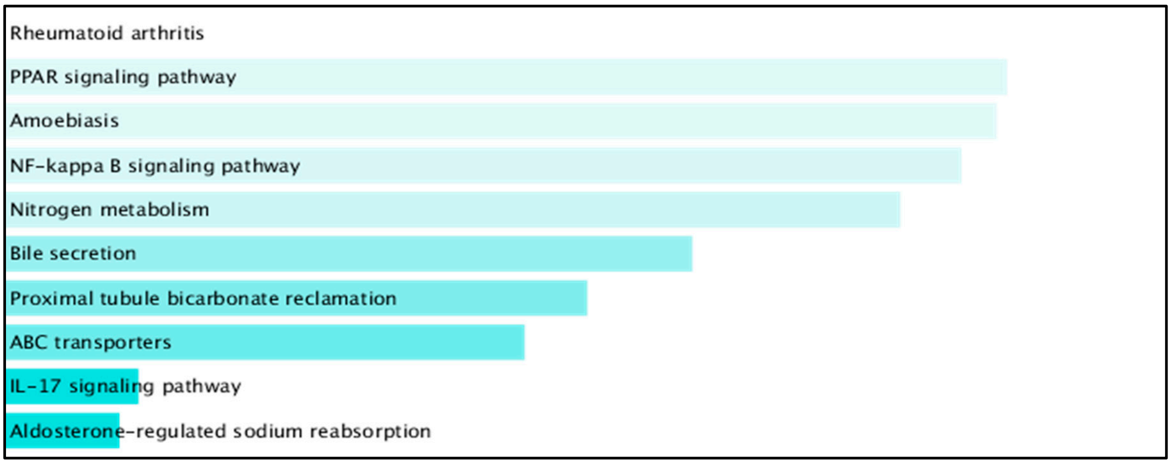

Figure S6. KEGG pathways of the Differentially Expressed Genes.

(A)

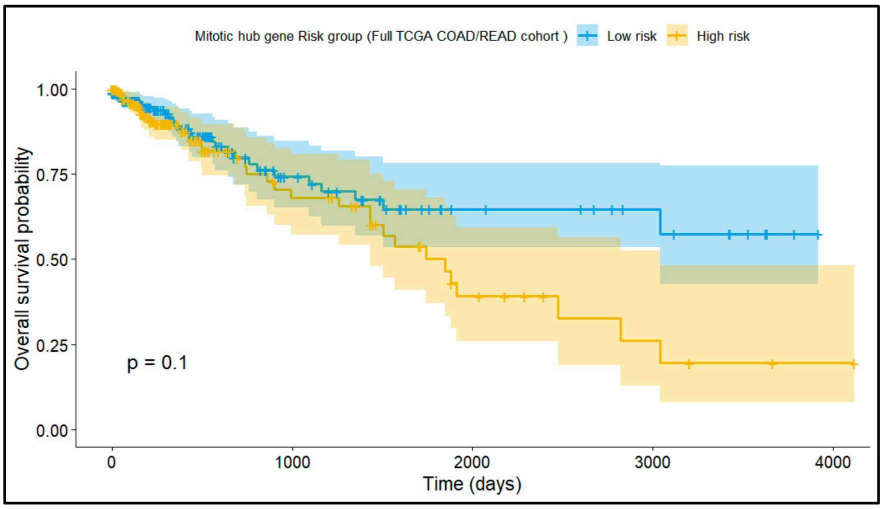

(B)

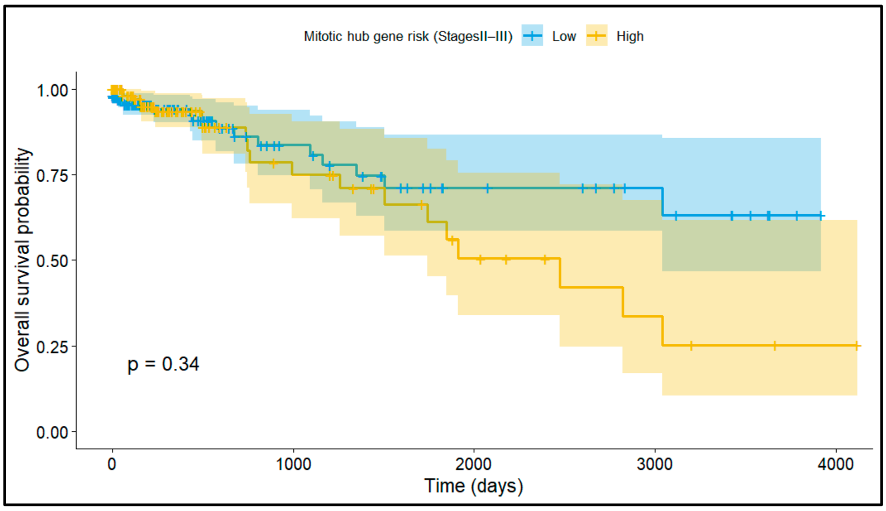

Figure S7. Overall survival according to mitotic hub gene risk group in the TCGA-COAD/READ cohort (A), stage II–III subset (B).

Kaplan–Meier overall survival curves comparing patients in the low-risk and high-risk groups defined by the mitotic hub gene signature in the full and stage II-III TCGA COAD/READ cohort. The blue curve represents the low-risk group, and the yellow curve represents the high-risk group. The shaded regions around each survival curve represent the 95% confidence intervals for the estimated survival probabilities. The log-rank test p-value is shown on the plot.

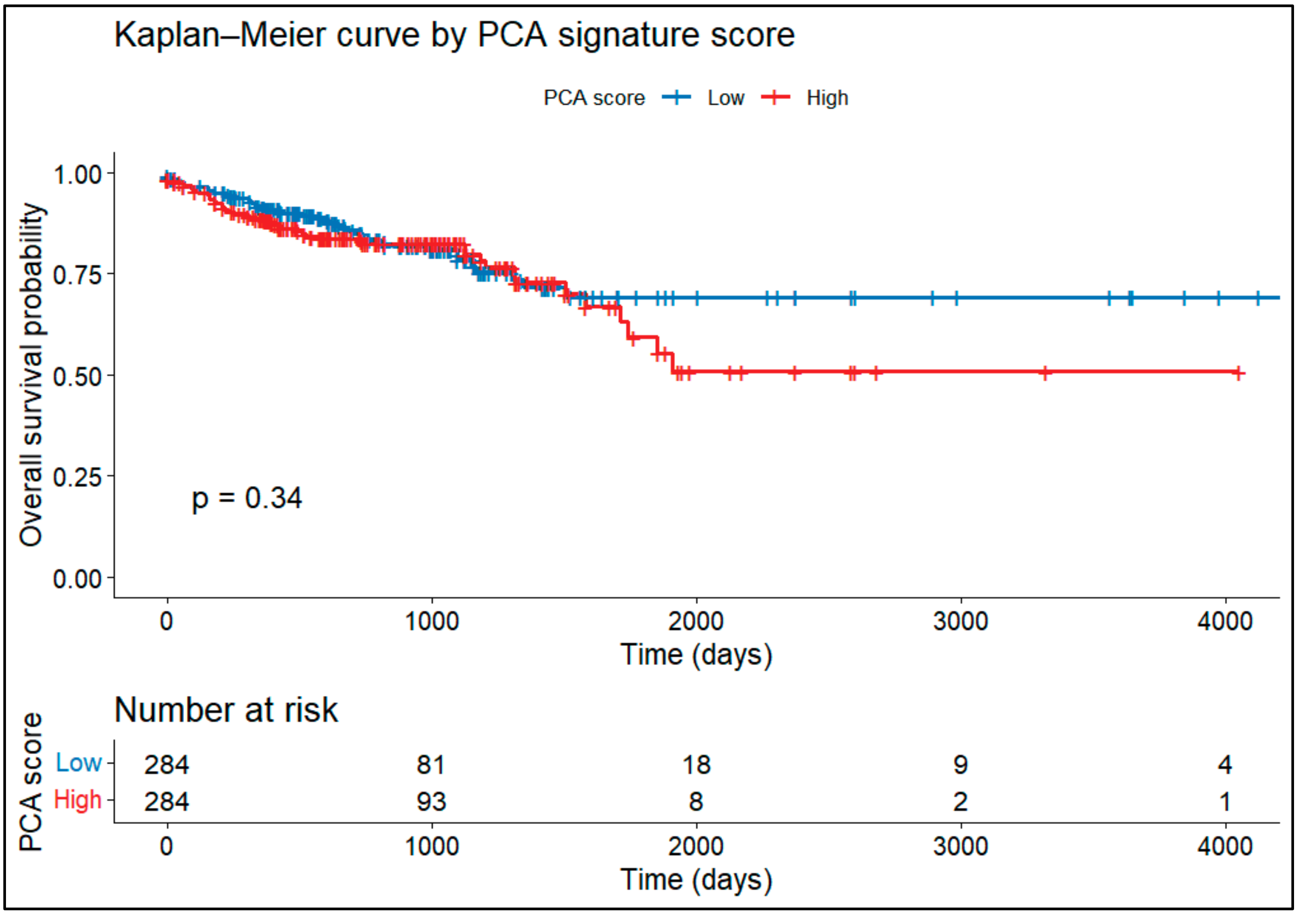

Figure S8. Kaplan- Meier curve by PCA signature score.

(A)

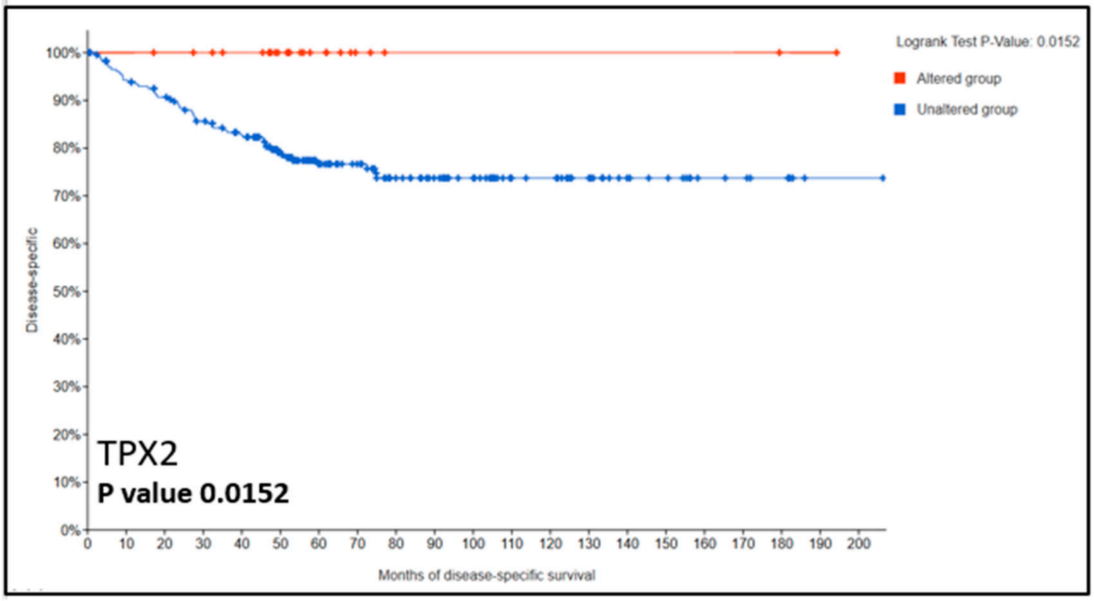

(B)

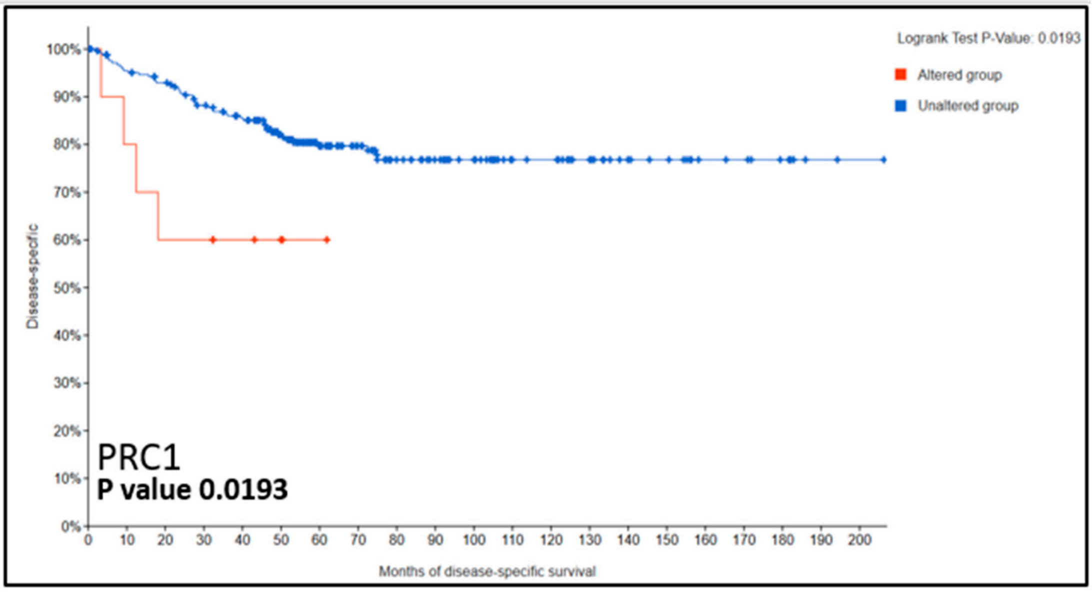

Figure S9. Disease specific survival of *TPX2* (A), *PRC1* (B), using Kaplan–Meier analysis

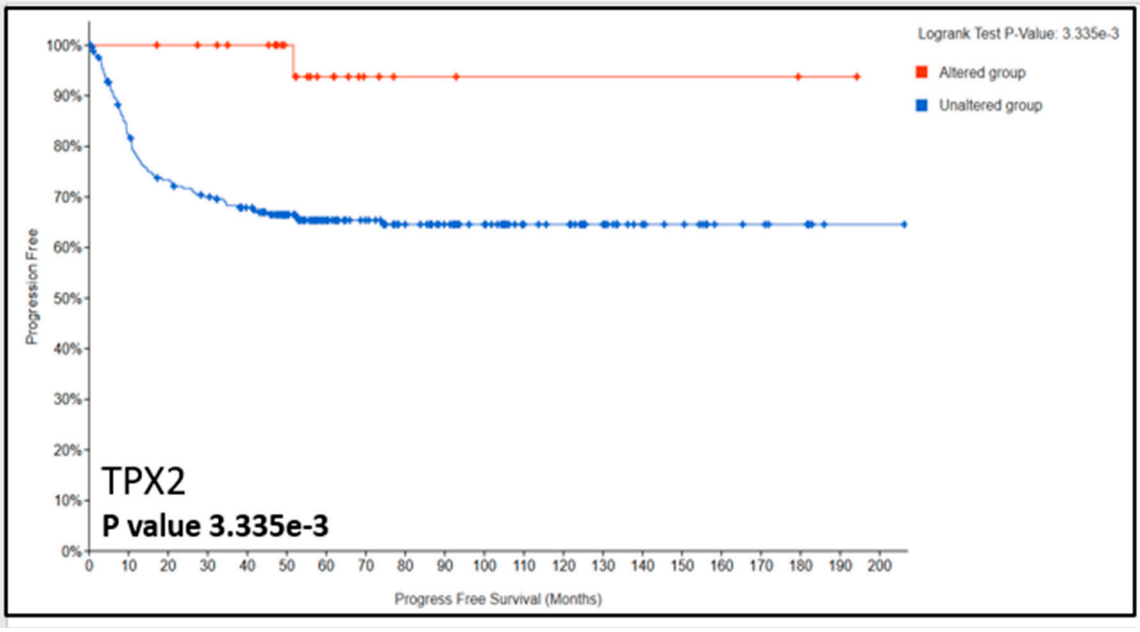

Figure S10.Prgression free survival of *TPX2* gene, using Kaplan–Meier analysis.

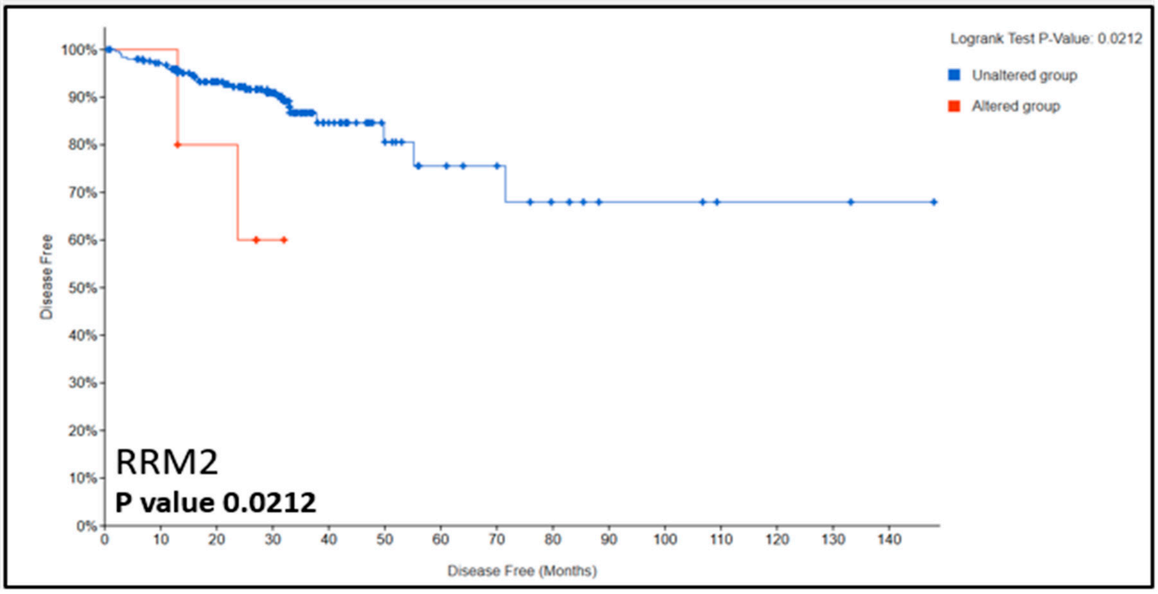

Figure S11.disease free survival of RRM2, using Kaplan–Meier analysis.

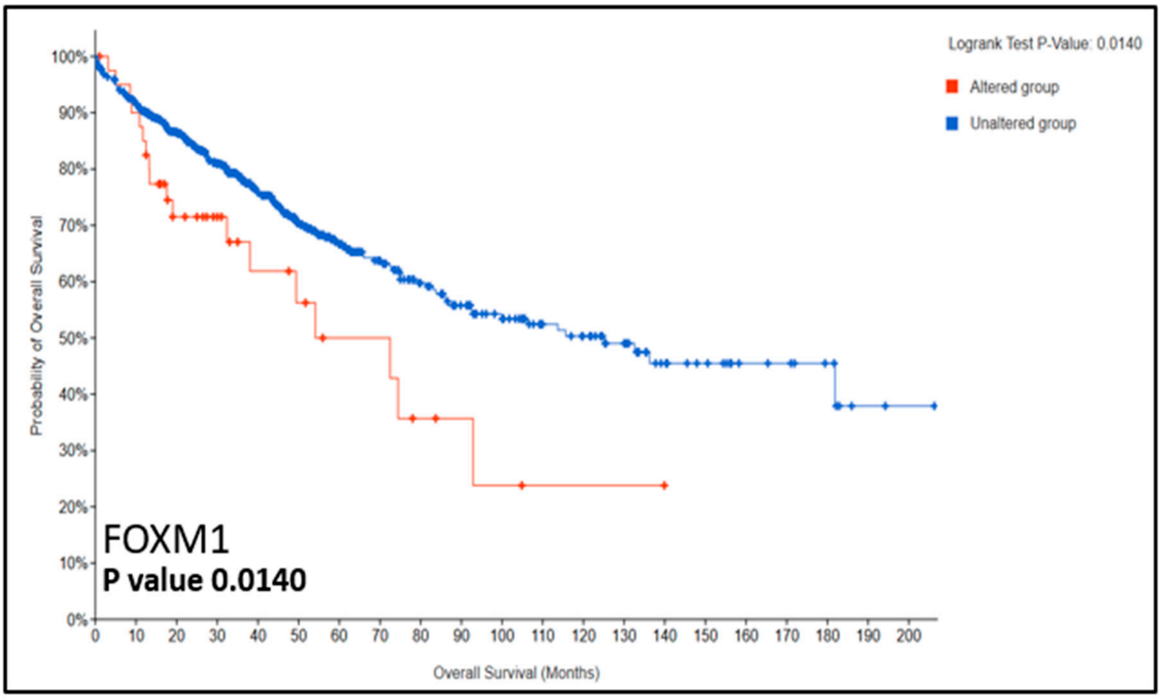

Figure S12. Probability of overall survival of FOXM1using Kaplan–Meier analysis.
